# Expression of Microtubule-Associated Proteins in Relation to Prognosis and Efficacy of Immunotherapy in Non-Small Cell Lung Cancer

**DOI:** 10.3389/fonc.2021.680402

**Published:** 2021-10-01

**Authors:** Jieyan Luo, Qipeng Hu, Maling Gou, Xiaoke Liu, Yi Qin, Jiao Zhu, Chengzhi Cai, Tian Tian, Zegui Tu, Yijia Du, Hongxin Deng

**Affiliations:** ^1^ Department of Thoracic Oncology, West China Hospital, Sichuan University, Chengdu, China; ^2^ State Key Laboratory of Biotherapy, West China Hospital, Sichuan University, Chengdu, China; ^3^ State Key Laboratory of Biotherapy and Cancer Center, West China Hospital, Sichuan University and Collaborative Innovation Center for Biotherapy, Chengdu, China

**Keywords:** MAP, non-small cell lung cancer, prognosis, immunotherapy, The Cancer Genome Atlas (TCGA)

## Abstract

**Background:**

Microtubule-associated proteins (MAPs) have been considered to play significant roles in the tumor evolution of non-small cell lung cancer (NSCLC). Nevertheless, mRNA transcription levels and prognostic value of distinct MAPs in patients with NSCLC remain to be clarified.

**Methods:**

In this study, the Oncomine database, Gene Expression Profiling Interactive Analysis (GEPIA) database, and Human Protein Atlas were utilized to analyze the relationship between mRNA/protein expression of different MAPs and clinical characteristics in NSCLC patients, including tumor type and pathological stage. The correlation between the transcription level of MAPs and overall survival (OS) of NSCLC patients was analyzed by Kaplan–Meier plotter. Besides, 50 frequently altered neighbor genes of the MAPs were screened out, and a network has been constructed *via* the cBioPortal and Search Tool for the Retrieval of Interacting Genes/Proteins (STRING) dataset. Meanwhile, we performed Gene Ontology (GO) and Kyoto Encyclopedia of Genes and Genomes (KEGG) pathway analysis on the expression data of MAPs and their 50 frequently altered neighbor genes in NSCLC tissues. Furthermore, The Cancer Immunome Atlas (TCIA) was utilized to analyze the relationship between MAP expression and the response to immunotherapy. Finally, we used reverse transcription-quantitative polymerase chain reaction (RT-qPCR) to verify the expression of MAPs in 20 patients with NSCLC.

**Results:**

The present study discovered that the mRNA transcription levels of MAP7/7D2 were enriched in NSCLC tissues, while those of the MAP2/4/6/7D3 were lower in NSCLC specimens than those in control specimens. The mRNA transcription level of MAP6 was significantly associated with the advanced stage of NSCLC. Besides, survival analysis indicated that higher mRNA expressions of MAP2/4/6/7/7D3 were correlated considerably with favorable OS of NSCLC patients, whereas increased mRNA expression levels of MAP1A/1S were associated with poor OS. Moreover, the expression of MAP1A/1B/1S/4/6/7D1/7D3 was significantly correlated with immunophenoscore (IPS) in NSCLC patients.

**Conclusions:**

Our analysis indicated that MAP1A/1S could serve as potential personalized therapeutic targets for patients with NSCLC, and the enriched MAP2/4/6/7/7D3 expression could serve as a biomarker for favorable prognosis in NSCLC. Besides, the expression of MAP1A/1B/1S/4/6/7D1/7D3 was closely related to the response to immunotherapy. Taken together, MAP expression has potential application value in the clinical treatment and prognosis assessment of NSCLC patients, and further verifiable experiments can be conducted to verify our results.

## Introduction

Non-small cell lung cancer (NSCLC), a common pathological type of lung cancer (LC), is one of the leading causes of cancer-related death worldwide (1, 2). NSCLC includes lung squamous cell carcinoma (LUSC), lung adenocarcinoma (LUAD), and large cell carcinoma. Meanwhile, patients with NSCLC account for approximately 85% of all LC patients (2). Although there has been substantial advancement in early screening and personalized treatment, the 5-year overall survival (OS) rate of LC remained at 21.2% in the United States (3). Hence, the underlying pathogenesis, prognostic markers, and equivalent targets of NSCLC should be understood and identified to enhance individualized therapeutic methods and associated prognosis. The changes in specific protein-related genes, such as mutations, translocations, deletions, and insertions, may assist in cancer development and tumor genetic regulation. In reality, some studies have demonstrated that microtubule-associated proteins (MAPs) are abnormally expressed in a variety of tumors, such as glioma (4), leukemia (5), and NSCLC (6).

MAP family, a series of proteins that were initially discovered to bind and stabilize microtubules, is generally classified into five groups based on their mode of function: (a) motile MAPs (7, 8), (b) depolymerase MAPs (9), (c) microtubule nucleated MAPs (10), (d) microtubule terminal-binding MAPs (11), and (e) structural MAPs. These MAP family members enhance the stability of microtubules, regulate the relationship between microtubules and other cellular components, and play pivotal roles in a variety of physiological processes, such as the spindle assembly and neuron formation (12, 13).

To date, more than 13 subtypes of MAPs have been recognized in mammalian cells. These proteins have been sequentially numbered, including MAP1A, MAP1B, MAP1S, MAP2, MAP4, MAP6, MAP7, MAP7D1, MAP7D2, MAP7D3 (14). Previous studies have found aberrant expressions and their prognostic value in some members of the MAP family. For instance, MAP4 was considerably overexpressed in LUAD clinical tissues and multiple cell lines (15). High expression of MAP4 was significantly associated with clinical and pathological stages of LUAD. At the same time, MAP2 is expressed explicitly in neuroendocrine carcinoma and relevant tumor cell lines, such as small cell lung cancer and neuroblastoma (15–17). Another study revealed that knockdown of MAP 1B-LC1 can decrease cell migration and invasion during epithelial–mesenchymal transition (EMT) in A549 cells (18). Nonetheless, the underlying mechanism by which MAP-related genes are regulated and the unique functions of MAP members in the development of NSCLC remain to be elucidated.

The relationship between abnormal expression levels of MAP family members and clinicopathologic staging and prognosis of NSCLC patients has been partially reported. Nevertheless, the roles of MAP members in the progression of NSCLC have not been analyzed using bioinformatics techniques. Hence, this study attempted to address this problem by analyzing the mRNA expression and mutations of different MAPs *via* microarray technology (19) and to identify the therapeutic potential personalized targets and prognostic value of MAPs for NSCLC patients. Meanwhile, the present study also determined the expected signaling pathways and corresponding functions of the MAP mutations as well as their 50 frequently altered neighbor genes.

## Materials and Methods

### Oncomine Analysis

Oncomine network station (http://www.oncomine.org/) is a tumor bioinformatics database that can provide services to DNA or RNA sequence analyses (20). In this study, transcriptional expressions of 10 different MAPs in diverse cancer tissues were analyzed *via* the Oncomine datasets. The MAP mRNA transcription levels of different cancer specimens were compared with those in corresponding control specimens. And the differences in the expression levels of MAP mRNA were compared by Student’s t-test. Cutoffs of p-value and fold change of expression levels were as follows: p-value: 0.01, fold change: 1.5, gene rank: 10%.

### Gene Expression Profiling Interactive Analysis Dataset

The GEPIA website (http://gepia.cancer-pku.cn/) is a network database which can provide analytical services for the mRNA transcriptional expressions of tumor or normal tissues derived from TCGA and other projects (21). The relationships between mRNA expression of different MAPs family members and the clinical data of NSCLC patients, which involve the tumor types and pathological stages, were analyzed in this GEPIA dataset. The mRNA expression plots in GEPIA were consistent with the Log2(TPM + 1) scale.

### Human Protein Atlas

The Human Protein Atlas website (https://www.proteinatlas.org) is a dataset that includes the various protein immunohistochemical patterns for common kinds of tumors and the corresponding different pathological types of these tumors (22). This network database can be utilized to identify specific mRNA/protein expression patterns in given tumors. In this study, the protein expression of different MAP members between human NSCLC and normal tissue specimens was compared directly by immunohistochemistry image.

### The Kaplan–Meier Plotter

The potential prognostic value of different MAP members’ transcription levels for patients with NSCLC was evaluated by an online database, Kaplan–Meier Plotter (http://kmplot.com/analysis/) (23). To analyze the correlation between the transcription levels of MAPs with OS of NSCLC patients, cancer specimens were split into two sets on account of median values of MAP mRNA expression (enriched and poor expression groups) and evaluated by Kaplan–Meier survival curves. In this study, the Kaplan–Meier survival plots include information on the hazard ratio (HR), 95% confidence intervals (CIs), and log-rank p-value that can be found in the Kaplan–Meier Plotter webpage. Additionally, the number-at-risk is revealed underneath the Kaplan–Meier survival patterns.

### The Cancer Genome Atlas and cBioPortal

The Cancer Genome Atlas (TCGA) (24), a comprehensive project aimed at the prevention of cancer ultimately, included gene sequencing data of diverse human tumors. By selecting Pan-Lung Cancer (TCGA, Nat Genet 2016) dataset containing genetic data from 1,144 case reports, the online tool cBioportal (25) (http://www.cbioportal.org/) was employed to analyze the mutation status of all MAPs in the Pan-Lung Cancer. Genomic profiles in the Pan-Lung Cancer (TCGA, Nat Genet 2016) dataset included somatic mutations and putative copy number alterations from genomic identification of significant targets in cancer (GISTIC).

### Extraction and Construction of Neighbor Gene Network

The Search Tool for the Retrieval of Interacting Genes/Proteins (STRING, https://string-db.org/) dataset, an interactive web server, is applicable to visualize, explore, and analyze the interrelationship between different proteins and equivalent genes (26). In this study, 50 frequently altered neighbor genes of the MAP family members were screened out, and a network has been constructed *via* the STRING dataset. This network of MAPs and neighbor genes provides valuable clues for analyzing the progress of NSCLC. This network pattern of MAPs and neighbor genes was constructed *via* the STRING website with the following setting: meaning of network edges: confidence; active interaction sources: text mining, experiments, databases, neighborhood; minimum required interaction score: 0.400; maximum number of interactors to show: 2nd shell–no more than 50 interactors.

### The Gene Ontology and Kyoto Encyclopedia of Genes and Genomes Analysis

Gene Ontology (GO) and Kyoto Encyclopedia of Genes and Genomes (KEGG) were utilized to analyze the expected signaling pathways and corresponding functions of MAP mutations and their 50 frequently altered neighbor genes *via* the Database for Annotation, Visualization, and Integrated Discovery (DAVID) dataset (27) (https://david.ncifcrf.gov/summary.jsp). Starting from the three directions of biological process (BP), cell component (CC), and molecular function (MF), the expected functions of target gene mutation can be predicted and analyzed by GO analysis. Meanwhile, KEGG tool was exploited to analyze MAP mutations and their 50 frequently altered neighbor genes and to identify the MAP-associated predictive pathways.

### The Cancer Immunome Atlas

The Cancer Immunome Atlas (TCIA; https://tcia.at/) is a dataset that contains TCGA data for 20 solid cancers with >8,000 tumor samples and can detect the immunophenoscore (IPS) of tumor samples, which can predict the response to cytotoxic T lymphocyte antigen-4 (CTLA-4) and programmed cell death protein 1 (PD-1) blockers (28). In this study, we got 1,037 IPSs of NSCLC samples *via* TCIA dataset. Meanwhile, NSCLC samples were divided into high and low expression groups according to the median value of MAPs/IPSs, respectively. In this way, we can analyze the relationships between MAPs and IPSs by the chi-square test and further clarify the correlations between MAPs and the response to immunotherapy.

### Tissue Collection

NSCLC tissues and adjacent non-tumor lung tissues were obtained from 20 patients (10 LUAD and 10 LUSC) who had undergone surgical resection of NSCLC during 2010–2013 in West China Hospital (WCH), Sichuan University, China. The patients were diagnosed with NSCLC based on histopathological evaluation. No treatment was performed preoperatively. All tissue samples were immediately snap-frozen in liquid nitrogen and then stored at −80°C until RNA extraction. The non-tumor tissue was located 5 cm from the edge of the tumor. According to the pathologist, no significant tumor cells were found in these areas. The study was approved by the Research Ethics Committee of WCH, Sichuan University, China.

### RNA Extraction and Reverse Transcription-Quantitative Polymerase Chain Reaction Analyses

Total RNA was extracted from tissues with the TRIzol reagent (Invitrogen, USA) according to the instructions. A reverse transcription kit (Takara, China) was used for cDNA synthesis. The reverse transcription-quantitative polymerase chain reaction (RT-qPCR) analysis was performed using a standard protocol from Power SYBR Green (Takara, China). The expression of MAPs was normalized using glyceraldehyde-3-phosphate dehydrogenase (GAPDH) as reference. The primers were synthesized by Tsingke (Chengdu, China). The primer sequences used in the studies are shown in **Supplementary Table S1**. The relative expression level of MAPs was calculated using 2^-ΔΔCt^ method and normalized by log2.

## Results

### Transcriptional Levels of Diverse Family Members of Microtubule-Associated Proteins in Non-Small Cell Lung Cancer

**Figure 1:**
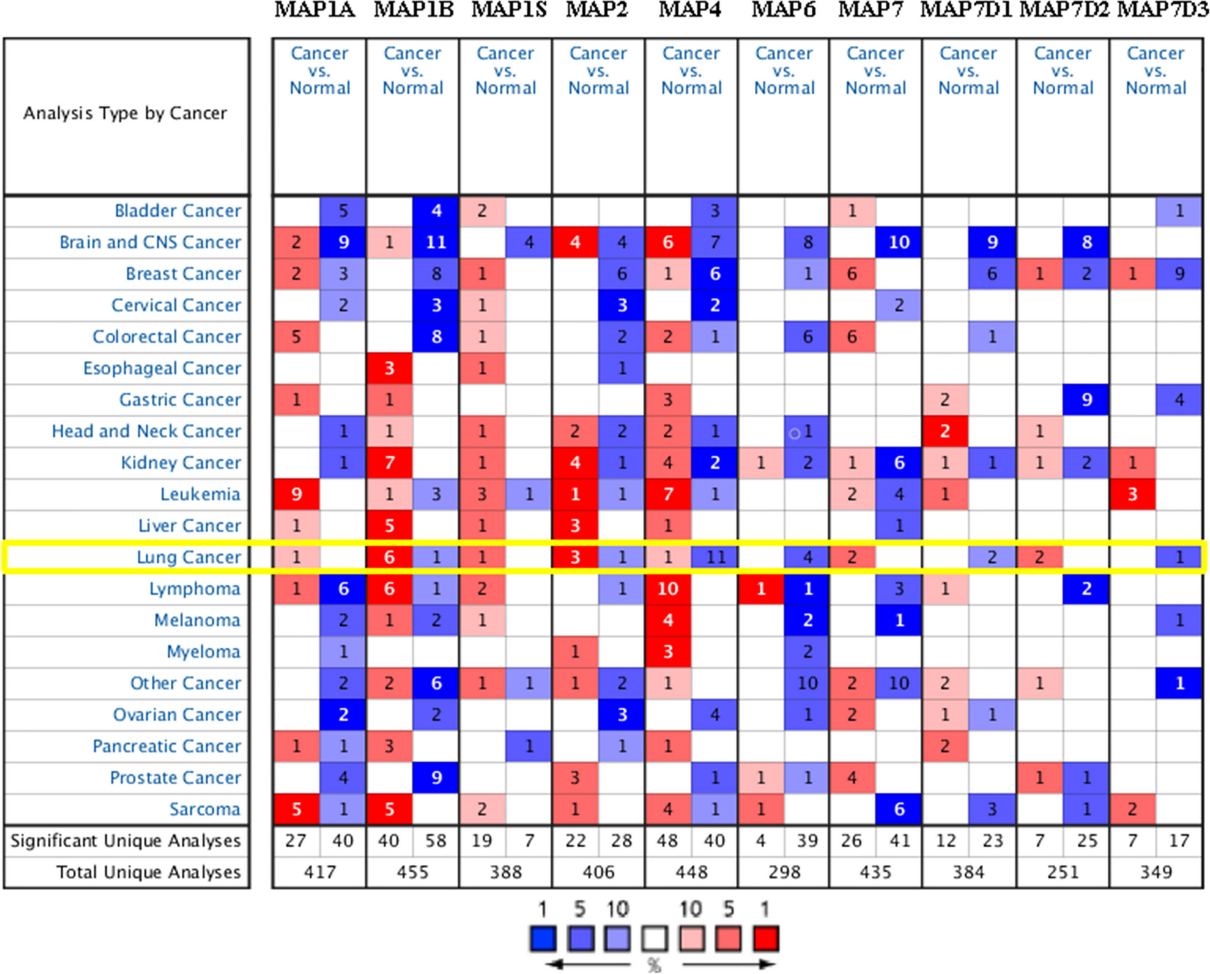
Transcriptional expression of MAPs in 20 types of tumors (Oncomine database). Differences in mRNA levels of MAPs were compared by Student’s t-test. Cutoffs of p-value and fold change of expression levels were as follows: p-value: 0.01, fold change: 1.5, gene rank: 10%. MAP, microtubule-associated protein.

In order to compare the mRNA transcriptional levels of different MAP members in tumors with those in control specimens, mRNA expression data were accessed and analyzed using the Oncomine database (www.oncomine.org). As shown in **Figure 1**, mRNA transcription levels of 10 members of MAPs in 20 types of tumors were retrieved and compared with those in normal tissues. Significantly higher mRNA expressions of MAP1A/1B/1S/2/7/7D2 were found in lung cancer specimens in numerous datasets (**Figure 1**). In this study, mRNA transcription levels of MAPs in NSCLC patients were our main observation object. In the Garber Lung dataset (29), MAP1B overexpression was found in LUSC specimens compared to control specimens with a fold change of 4.108 (p = 3.95E-4). Meanwhile, a 2.384-fold increase in MAP1B mRNA expression was observed in large cell lung carcinoma samples (p = 4E-3). Besides, the Hou Lung dataset (30) revealed a 3.136-fold increase in MAP1B mRNA expression in large cell lung carcinoma tissues (p = 9.06E-6) (**Table 1**). In the Selamat Lung dataset (31), MAP1S was enriched in LUAD with a 1.786-fold increase (p = 6.09E-17) (**Table 1**). Similarly, the Su Lung dataset (32) showed another mRNA expression with a boost; that is, MAP2 has a 1.620-fold increase in LUAD specimens compared with control specimens (p = 7.13E-4) (**Table 1**).

**Table 1:** Differences in transcriptional expression of diverse MAPs between NSCLC and normal lung specimens (Oncomine Database).

	Types of NSCLC *vs*. Lung	Fold Change	p-Value	Source and/or Reference
**MAP1B**				
	Squamous cell lung carcinoma	4.108	3.95E-4	Garber Lung (29)
	Large cell lung carcinoma	2.384	4E-3	Garber Lung (29)
	Large cell lung carcinoma	3.136	9.06E-6	Hou Lung (30)
**MAP1S**				
	Lung adenocarcinoma	1.786	6.09E-17	Selamat Lung (31)
**MAP2**				
	Lung adenocarcinoma	1.620	7.13E-4	Su Lung (32)
**MAP7**				
	Lung adenocarcinoma	1.706	1.25E-4	Su Lung (32)
**MAP7D2**				
	Large cell lung carcinoma	5.453	1.33E-6	Hou Lung (30)
	Lung adenocarcinoma	6.785	4.31E-11	Okayama Lung (33)

NSCLC, non-small cell lung cancer; MAP, microtubule-associated protein.

Moreover, the Su Lung dataset (32) showed a 1.706-fold increase in MAP7 mRNA expression in LUAD tissues (p = 1.25E-4) (**Table 1**). Significant upregulation of MAP7D2 was also found in NSCLC specimens compared to normal tissues. The result from the Hou Lung dataset (30) showed that there were 5.453-fold (p = 1.33E-6) in MAP7D2 mRNA expression in large cell lung carcinoma. In contrast, the Okayama Lung dataset (33) revealed a 6.785-fold increase in MAP7D2 mRNA expression in LUAD tissues (p = 4.31E-11) (**Table 1**).

### The Transcriptional Pattern of Microtubule-Associated Proteins in The Cancer Genome Atlas

**Figure 2:**
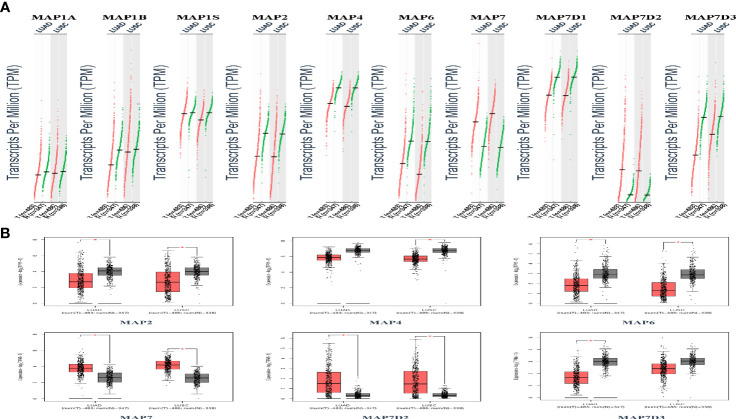
Transcription levels of distinct MAPs in LUAD, LUSC, and normal lung specimens (GEPIA). **(A)** Scatter diagram. **(B)** Box plot. *p < 0.05. GEPIA, Gene Expression Profiling Interactive Analysis; LUAD, lung adenocarcinoma; LUSC, lung squamous cell carcinoma; MAP, microtubule-associated protein.

**Figure 3:**
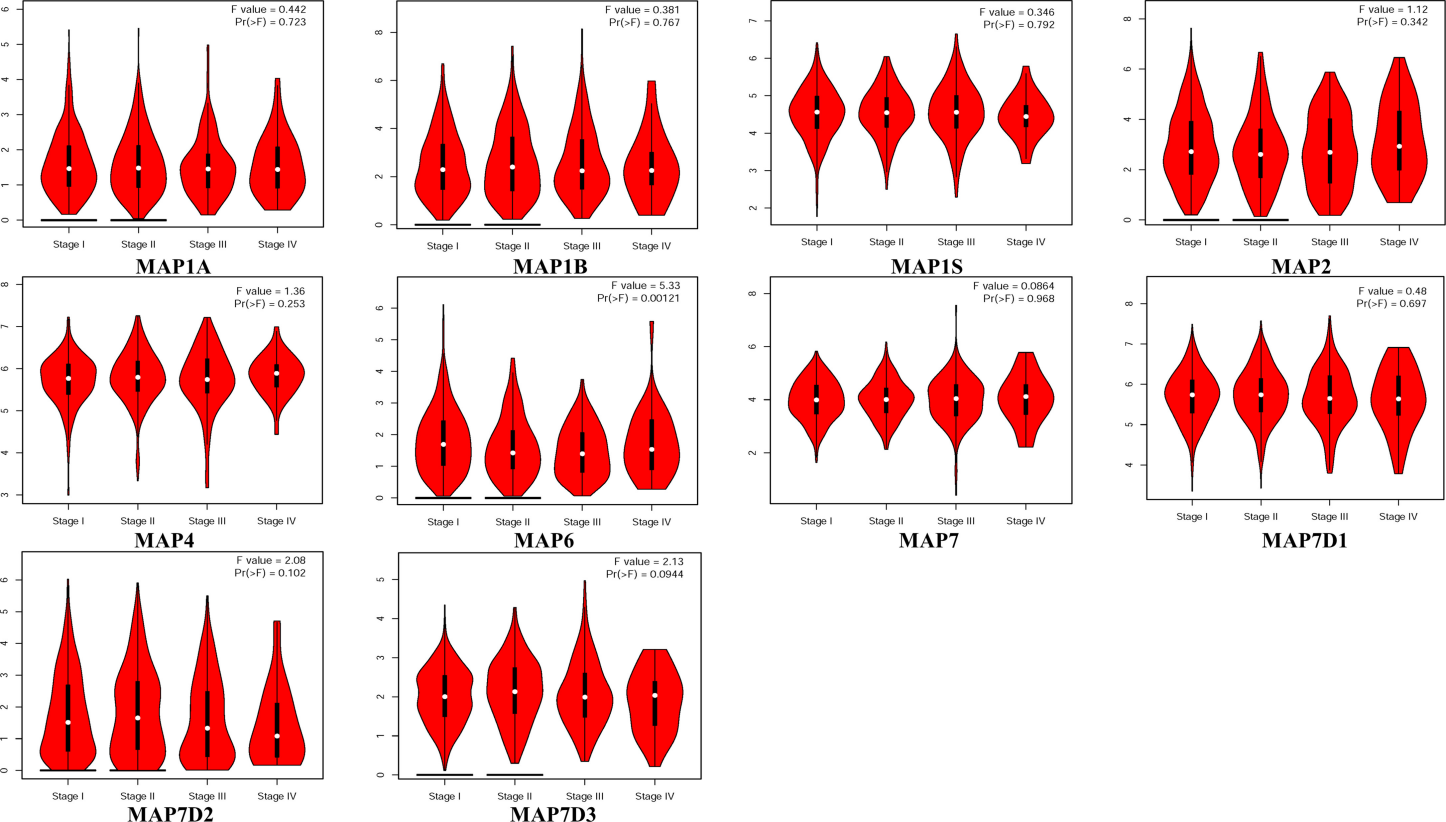
Correlation between MAP expression and cancer stage in NSCLC (GEPIA). GEPIA, Gene Expression Profiling Interactive Analysis; MAP, microtubule-associated protein; NSCLC, non-small cell lung cancer.

GEPIA is a newly developed interactive web server for analyzing the RNA sequencing expression data of 9,736 tumors and 8,587 normal samples from TCGA and the GTEx projects using a standard processing pipeline. In order to explore potential personalized therapeutic targets and prognostic value of different MAP members in NSCLC tissues with those in normal specimens, mRNA and protein expression data were accessed and analyzed by Gene Expression Profiling Interactive Analysis (GEPIA) dataset and Human Protein Atlas (https://www.proteinatlas.org). Firstly, utilizing the GEPIA, transcription levels of different MAPs between LUAD, LUSC, and normal lung specimens were compared. The results indicated that the transcription levels of MAP7/7D2 were higher in LUAD and LUSC specimens than those in normal lung specimens, while those of MAP2/4/6/7D3 were just the opposite (**Figure 2**). MAP2/4/6/7/7D2/7D3 groups significantly varied, whereas MAP1A/1B/1S/7D1 groups did not significantly differ (**Supplementary Figure S1**). Furthermore, the transcription levels of the MAP4 group in LUAD tissues and the MAP7D3 group in LUSC tissues were not significantly different from those in normal lung specimens (**Figure 2**). Besides, transcription levels of different MAPs with clinical cancer stage were also analyzed for LUSC and LUAD. The results indicated that the transcription level of MAP6 in NSCLC was significantly varied and correlated with advanced tumor stage (**Figure 3**).

**Figure 4:**
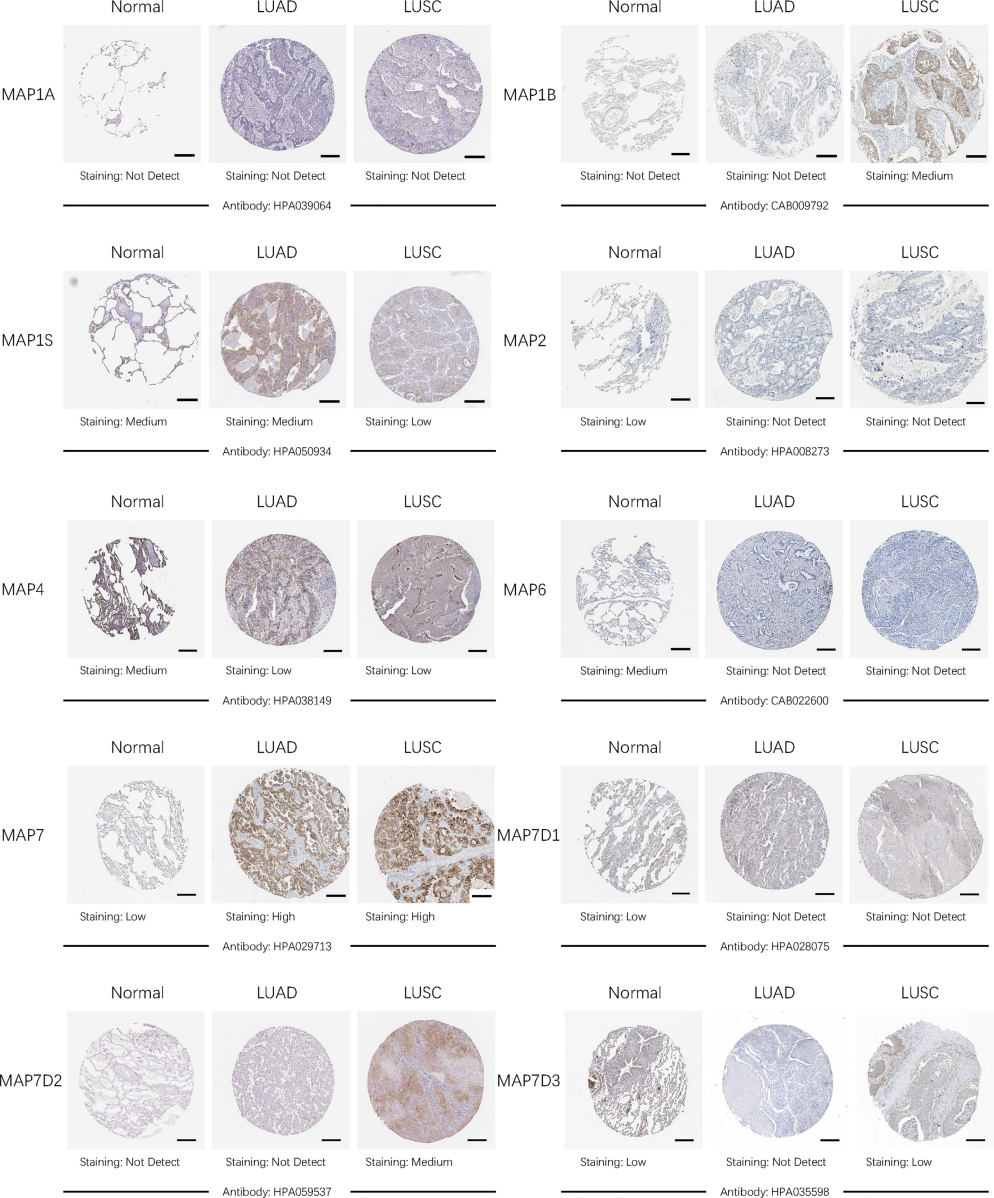
Representative immunohistochemistry images of distinct MAPs in cancer and paratumor tissues (Human Protein Atlas). MAP, microtubule-associated protein.

After comparing the transcription levels of MAPs in LUAD, LUSC, and normal lung specimens, in the present study, Human Protein Atlas was implemented to examine and measure the protein expression levels of MAPs in LUAD, LUSC tissues, and normal lung specimens. MAP1A protein was not expressed in LUAD, LUSC, and normal lung specimens, as diagrammed in **Figure 4**. Meanwhile, MAP1B/7D2 proteins were not observed to be expressed in normal lung specimens, while medium expression was shown in LUSC specimens (**Figure 4**). Similarly, low protein expression of MAP7 was observed in normal lung specimens, while high protein expression was demonstrated in LUAD and LUSC specimens (**Figure 4**). Furthermore, higher protein expression of MAP1S/2/4/6/7D1/7D3 was expressed in normal lung specimens, while lower protein expression was observed in LUAD or/and LUSC tissues (**Figure 4**). Taken together, the results derived from the Human Protein Atlas dataset showed that transcriptional and proteinic expression levels of MAP1B/7/7D2 were more enriched in LUAD or/and LUSC specimens than those in normal lung specimens, while those of MAP1S/2/4/6/7D1/7D3 were just the opposite.

### Prognostic Value of Diverse Microtubule-Associated Proteins in Non-Small Cell Lung Cancer

**Figure 5:**
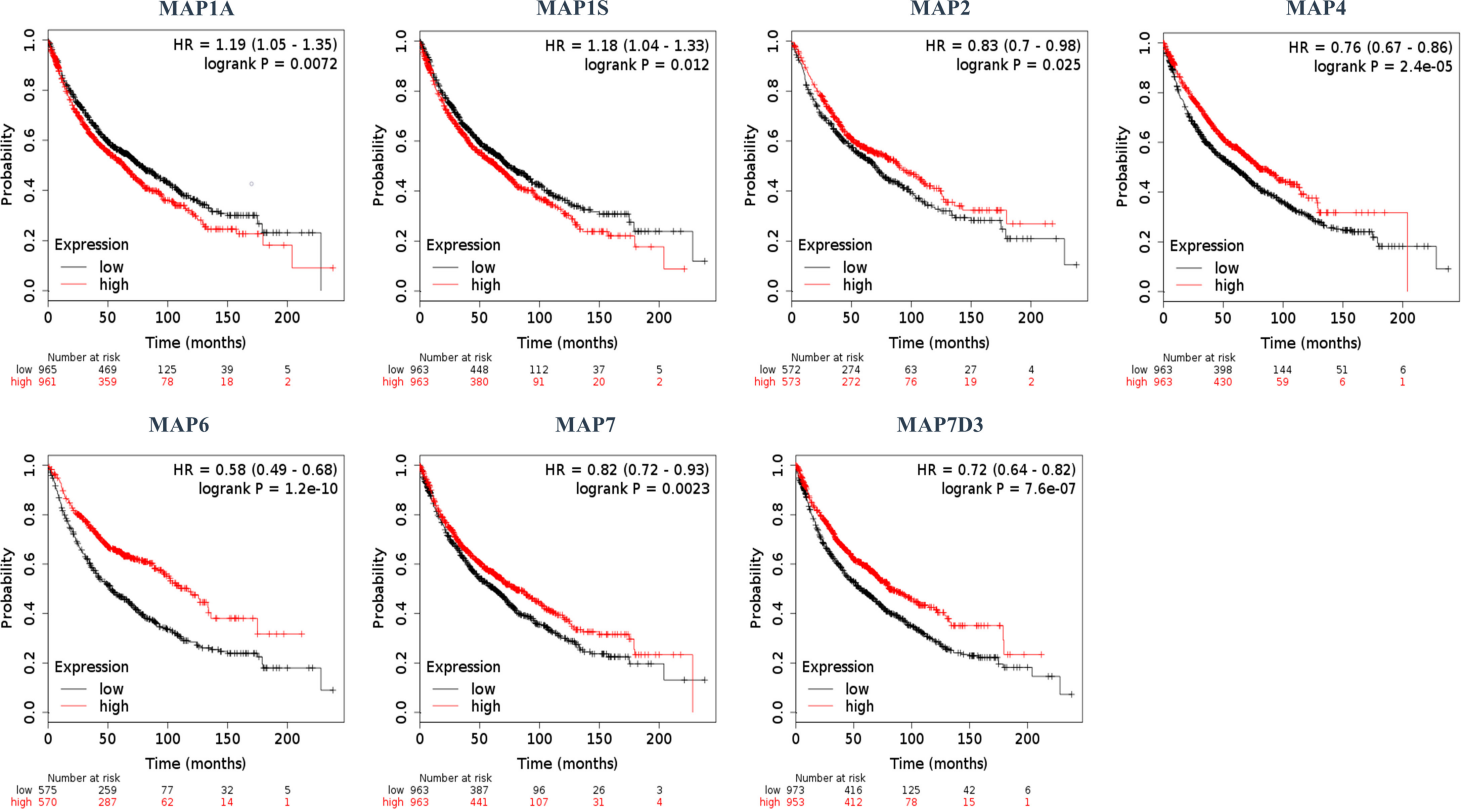
Kaplan–Meier survival curves stratified by seven MAPs, respectively. MAP, microtubule-associated protein.

Furthermore, the Kaplan–Meier plotter was applicable to analyze the correlation between mRNA transcription levels of MAPs and patient prognosis in NSCLC. As **Figure 5** shows, most MAPs were significantly correlated with the prognosis of NSCLC. The analysis plots revealed that higher mRNA expression of MAP1A/1S was significantly correlated with shorter OS of NSCLC patients, while that of MAP2/4/6/7/7D3 was just the opposite (**Figure 5**). Besides, MAP1B/7D1/7D2 mRNA expression levels showed no significant correlation with prognosis of NSCLC patients (**Supplementary Figure S2**). The above results revealed that the mRNA expression levels of MAP1A/1S/2/4/6/7/7D3 were significantly associated with the prognosis of NSCLC patients, and they might be utilized as possible prognostic markers in NSCLC patients.

### Expected Signaling Pathways and Corresponding Functions of the Microtubule-Associated Protein Mutations and Their Frequently Altered Neighbor Genes in Non-Small Cell Lung Cancer

**Figure 6:**
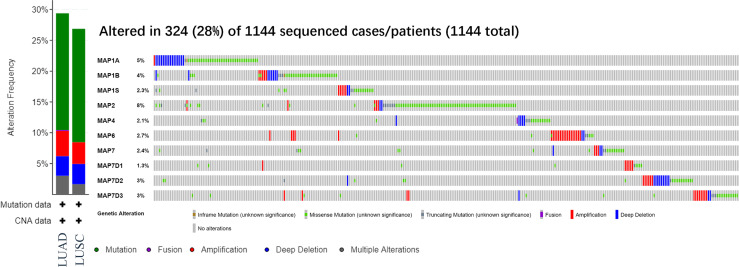
MAP gene alterations analysis in LUAD and LUSC (cBioPortal). LUAD, lung adenocarcinoma; LUSC, lung squamous cell carcinoma; MAP, microtubule-associated protein.

**Figure 7:**
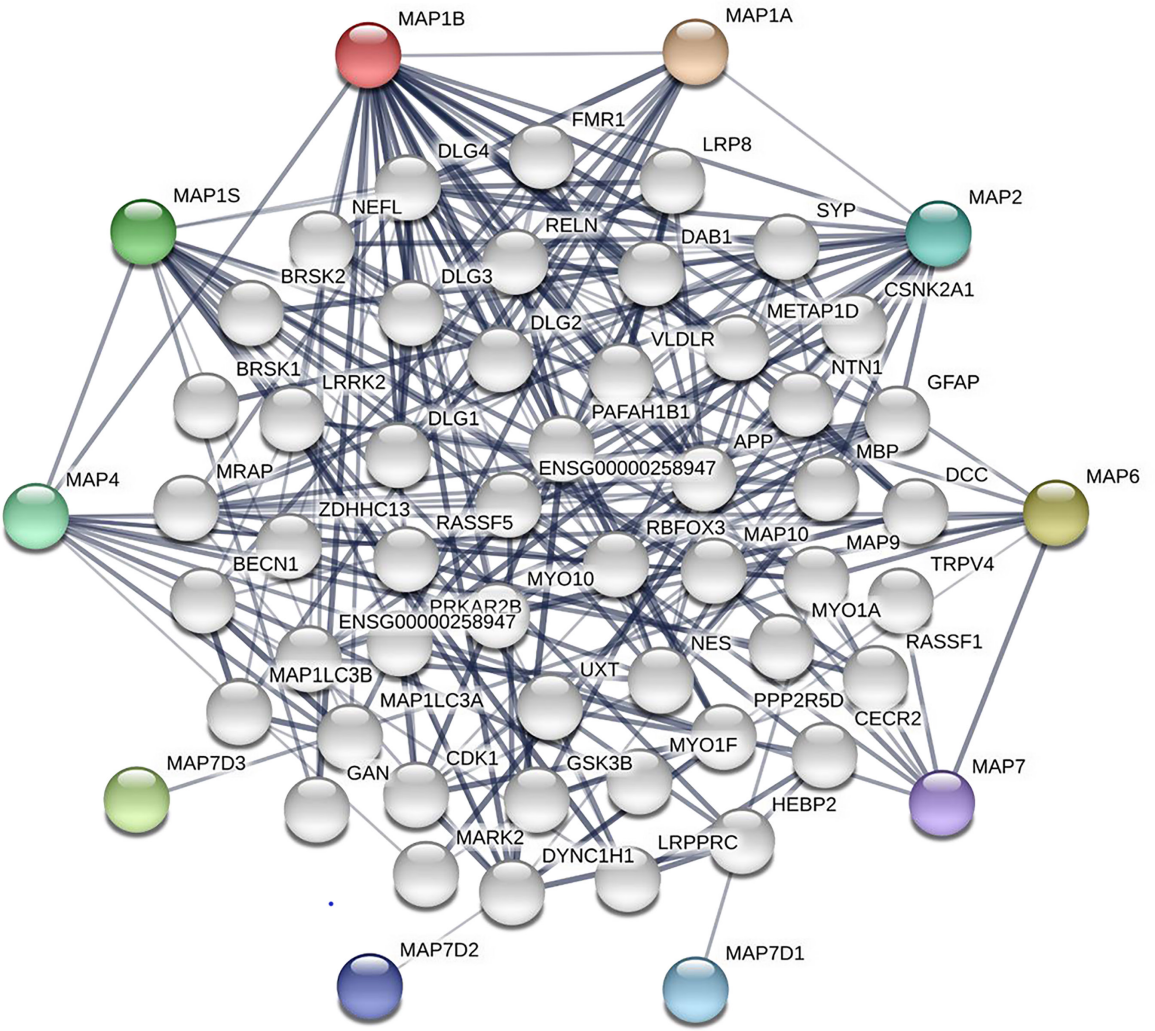
Network of MAP mutations and their 50 frequently altered neighbor genes in NSCLC (STRING). MAP, microtubule-associated protein; NSCLC, non-small cell lung cancer; STRING, Search Tool for the Retrieval of Interacting Genes/Proteins.

After analyzing the prognostic value of different MAPs in NSCLC, the MAP alterations were analyzed by utilizing the cBioPortal dataset (www.cbioportal.org) for NSCLC (**Figure 6**). The results showed that MAP alterations were present in 324/1144 NSCLC patients (28%) (**Figure 6**). Next, 50 frequently altered neighbor genes, which were significantly correlated with MAP mutations, and associated networks were further analyzed and constructed *via* the STRING dataset (**Figure 7**). The results showed that the Hippo signaling pathway-related genes, including DLG1, DLG2, DLG3, DLG4, RASSF1, and GSK-3β, were significantly associated with MAP alterations (**Figure 7**).

**Figure 8:**
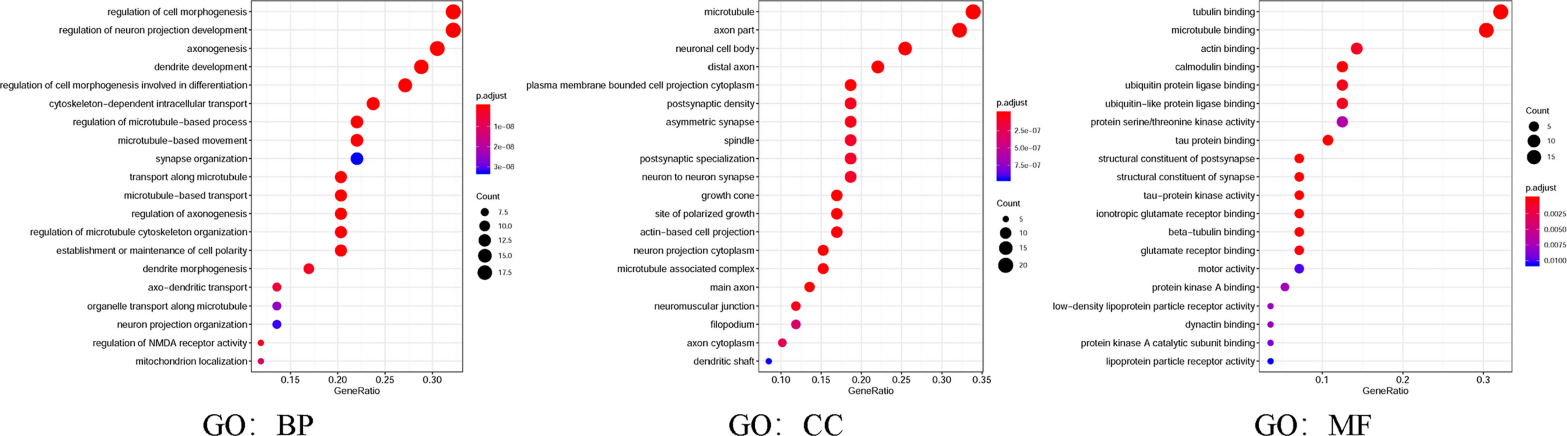
Gene Ontology (GO) was utilized to analyze the expected functions of MAP mutations and their 50 frequently altered neighbor genes *via* the DAVID dataset. Starting from the three directions of biological processes (GO: BP), cell components (GO: CC), and molecular functions (GO: MF), the expected functions of target gene mutation can be predicted and analyzed. DAVID, Database for Annotation, Visualization, and Integrated Discovery; MAP, microtubule-associated protein.

Moreover, in the present study, GO and KEGG were utilized to analyze the expected signaling pathways and corresponding functions of MAP mutations and their 50 frequently altered neighbor genes *via* the DAVID dataset. Proceeding from the three directions of BP, CC, and MF, the expected functions of target gene mutation can be predicted and analyzed by GO analysis (**Figure 8**). The BPs such as GO: 0022604 (regulation of cell morphogenesis), GO: 0010975 (regulation of neuron projection development), GO: 0007409 (axonogenesis), GO: 0016358 (dendrite development), and GO: 0010769 (regulation of cell morphogenesis involved in differentiation) were remarkably regulated by the MAP mutations in NSCLC (**Figure 8**). The CCs, including GO: 0005874 (microtubule), GO: 0033267 (axon part), GO: 0043025 (neuronal cell body), and GO: 0150034 (distal axon) were likewise significantly associated with the MAP alterations (**Figure 8**). Also, MAP mutations prominently affected the MFs, such as GO: 0015631 (tubulin binding), GO: 0008017 (microtubule binding), and GO: 0003779 (actin binding) (**Figure 8**).

**Figure 9:**
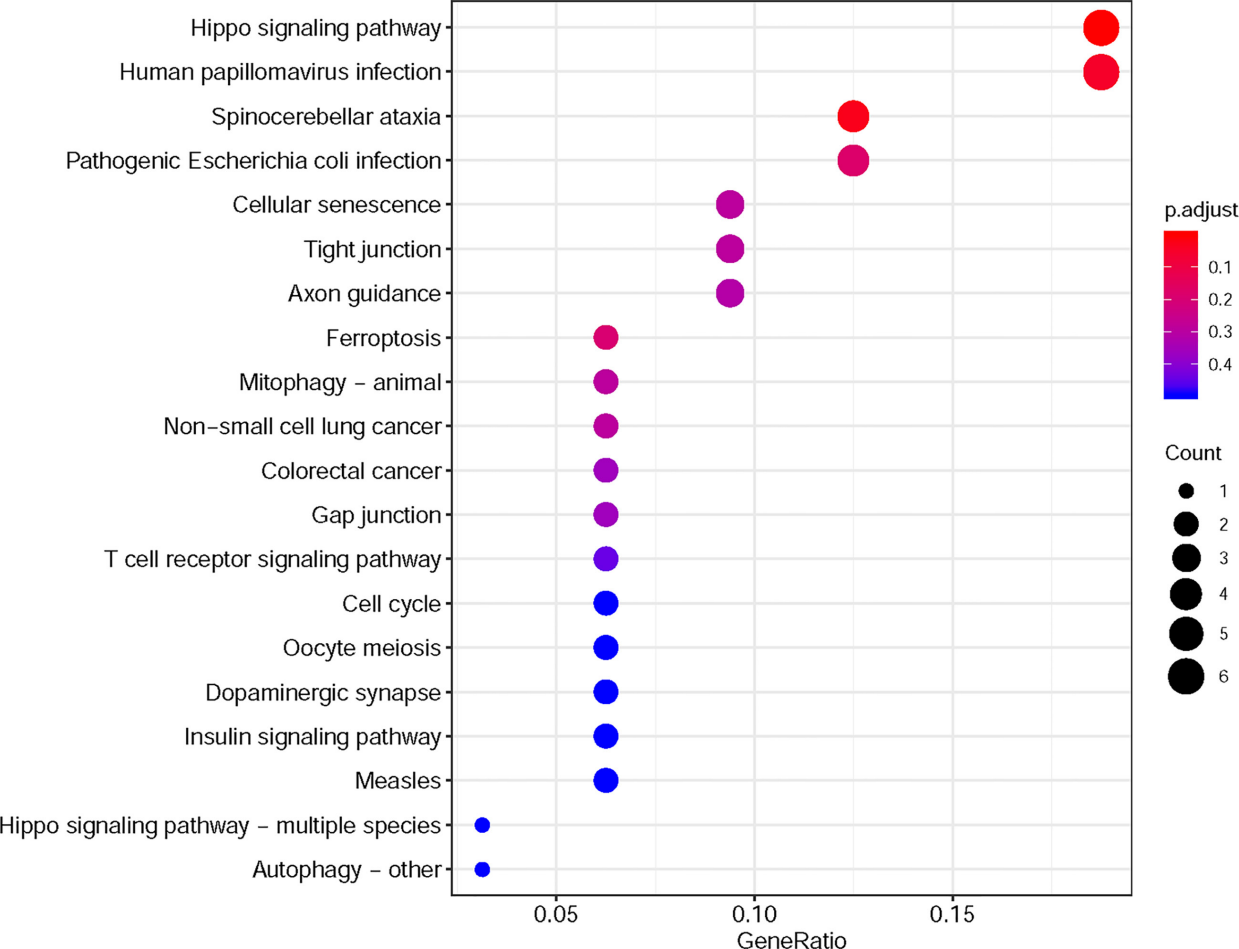
KEGG pathway analysis tool was utilized to analyze the predicted pathways of MAP mutations and their 50 frequently altered neighbor genes *via* the DAVID dataset. DAVID, Database for Annotation, Visualization, and Integrated Discovery; KEGG, Kyoto Encyclopedia of Genes and Genomes; MAP, microtubule-associated protein.

**Figure 10:**
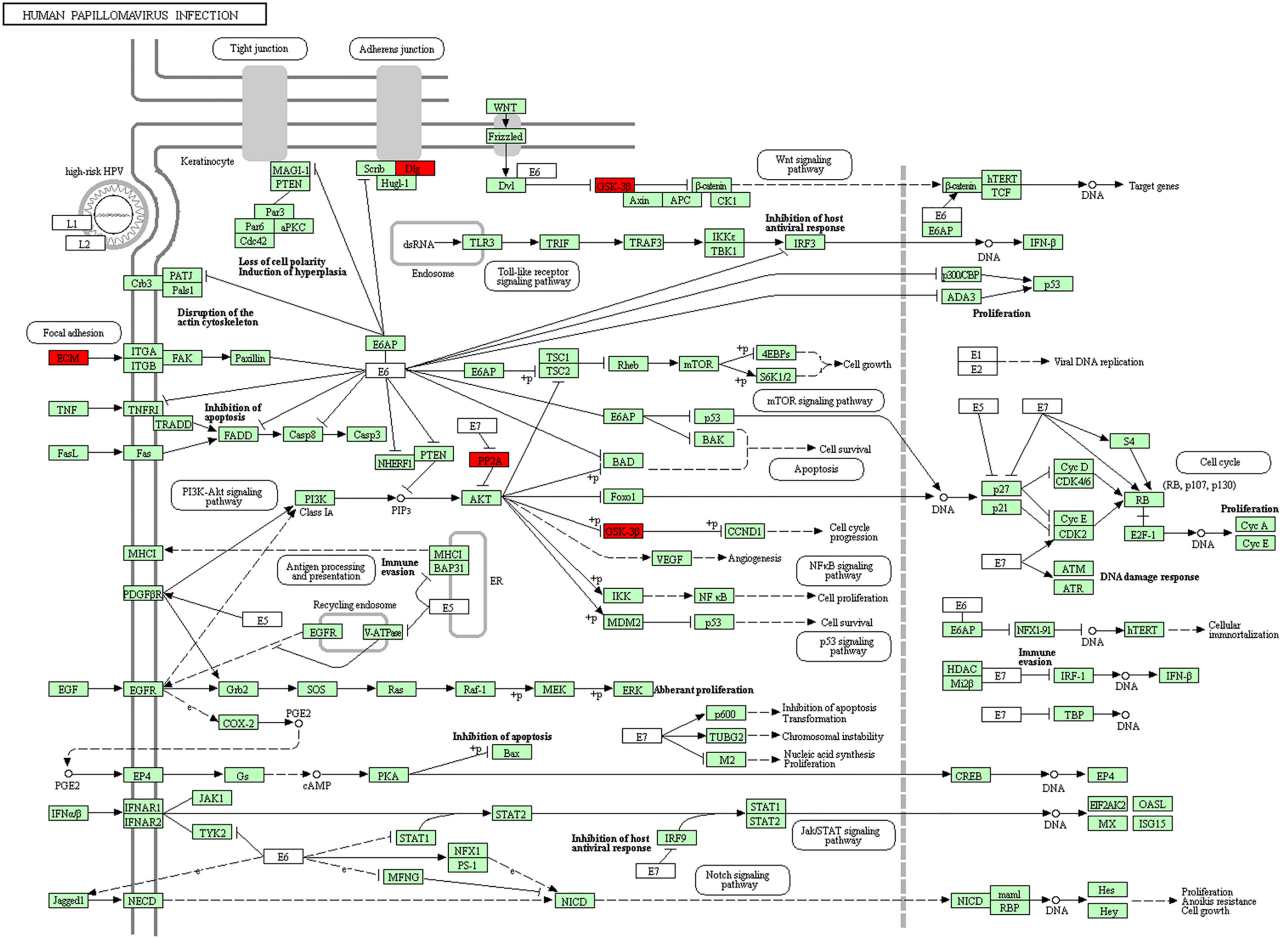
Human papillomavirus infection signal pathway regulated by MAP mutations of NSCLC patients (DAVID). DAVID, Database for Annotation, Visualization, and Integrated Discovery; MAP, microtubule-associated protein; NSCLC, non-small cell lung cancer.

Furthermore, in this study, we exploited the KEGG tool to analyze MAP mutations and their 50 frequently altered neighbor genes and to identify the MAP-related predictive pathways (**Figure 9**). The analysis result indicated that three pathways including has: 04390 (Hippo signaling pathway), has: 05017 (Spinocerebellar ataxia), and has: 05165 (Human papillomavirus infection) were associated with the functions of MAP mutations in NSCLC (**Figure 9**). As shown in **Figure 10**, the human papillomavirus infection signal pathway regulated by MAP mutations of NSCLC patients can be predicted using KEGG analysis.

### Microtubule-Associated Protein Expression Was Significantly Correlated With Response to Immunotherapy in Non-Small Cell Lung Cancer

Tumor-infiltrating lymphocytes were different in the high and low expression groups of MAPs (**Supplementary Figure S3**). In order to determine the relationship between MAPs and the response to CTLA-4 and PD-1 blockers, firstly, we calculated the IPS of 1,037 NSCLC samples exported from TCGA database by TCIA (https://tcia.at/) database (**Supplementary Material S1** and **Supplementary Table S2**). Then, the 1,037 NSCLC samples were divided into high and low expression groups according to the median value of MAPs/IPS (**Supplementary Tables S2, S3**), respectively. Next, the relationships between MAPs and IPS were analyzed by the chi-square test. As **Table 2** shows, the expression levels of MAP1A/1B/1S/4/6/7D1/7D3 were significantly correlated with IPS in NSCLC patients. The higher the IPS, the better the response to CTLA-4 and PD-1 blockers (28). Thus, we can conclude that the expression levels of MAP1A/1B/1S/4/6/7D1/7D3 were closely related to the response to immunotherapy.

**Table 2:** Correlations between MAPs and IPS (chi-square test).

	Gene expression	Cases	IPS-High	IPS-Low	p-Value
**MAP1A**	High	518	320	198	0.004480 *
	Low	519	365	154	
**MAP1B**	High	518	310	208	0.000033 *
	Low	519	375	144	
**MAP1S**	High	518	364	154	0.005147 *
	Low	519	321	198	
**MAP2**	High	518	349	169	0.406379
	Low	519	336	183	
**MAP4**	High	518	317	201	0.001213 *
	Low	519	368	151	
**MAP6**	High	518	373	145	0.000069 *
	Low	519	312	207	
**MAP7**	High	518	342	176	0.965447
	Low	519	343	176	
**MAP7D1**	High	518	323	195	0.014336 *
	Low	519	362	157	
**MAP7D2**	High	518	342	176	0.965447
	Low	519	343	176	
**MAP7D3**	High	518	295	223	<0.000001*
	Low	519	390	129	

MAP, microtubule-associated protein; IPS, immunophenoscore. *p < 0.05.

### Reverse Transcription-Quantitative Polymerase Chain Reaction Results Agreed With Gene Expression Profiling Interactive Analysis: MAP7/7D2 Expression Was Higher in Non-Small Cell Lung Cancer Samples Than That in Paratumor Tissues, but MAP2/4/6/7D3 Expression Was the Opposite

**Figure 11:**
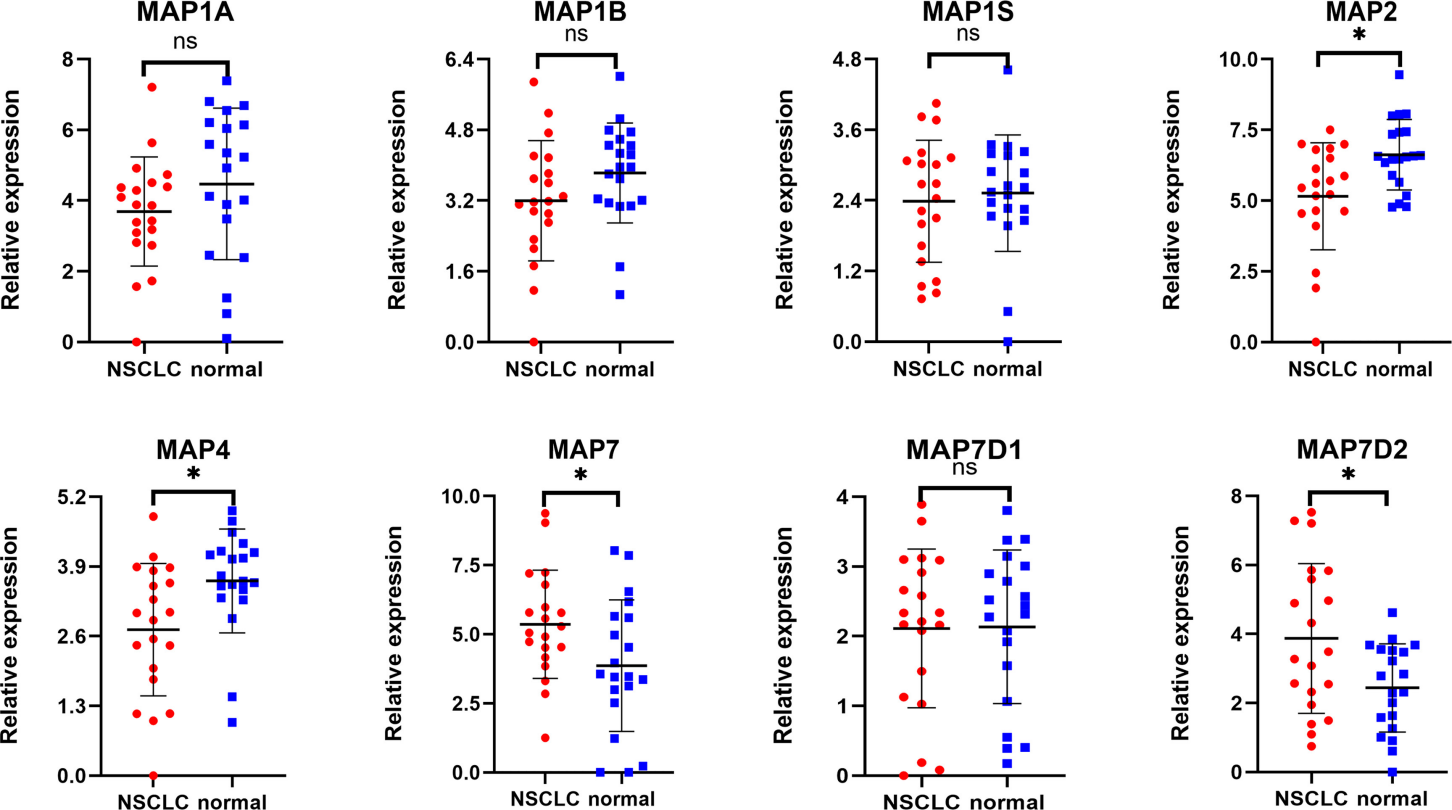
RT-qPCR analysis of MAP expression in 20 pairs of NSCLC samples and paratumor tissues. *p < 0.05. MAP, microtubule-associated protein; NSCLC, non-small cell lung cancer; RT-qPCR, reverse transcription-quantitative polymerase chain reaction. ns, not significant.

Expression profiles of MAPs in 20 pairs of NSCLC samples and paratumor tissues were evaluated using RT-qPCR that was performed by ourselves. Results are given below: MAP7/7D2 expression was higher in NSCLC samples than that in paratumor tissues, but MAP2/4 expression was the opposite. Besides, MAP1A/1B/1S/7D1 expression was similar between NSCLC samples and paratumor tissues (**Figure 11**; * p < 0.05). Similarly, MAP6/7D3 expression was also lower in NSCLC samples than in paratumor tissues, albeit with no statistical significance achieved (**Supplementary Figure S4**). All the above results are consistent with the analysis results of GEPIA.

## Discussion

Being important components to bind and stabilize microtubules in a variety of physiologies, such as the assembly of spindles during cell division in mammalian cells, MAP family members are implicated in the development of multiple cancers, including NSCLC. Although the MAP family has been ascertained to play critical roles in tumorigenesis and prognosis of a variety of tumors, further bioinformatics analysis of distinct roles of MAP family members in NSCLC remains to be performed. The present study explored the mRNA transcription levels, protein expression levels, and associated prognosis (OS) of diverse members of MAPs in NSCLC patients. Moreover, it is supposed that the findings of the present research might contribute to broadening current knowledge, improving the design of cancer treatments, and improving the accuracy of prognosis in patients with NSCLC.

Results from this research indicated that enriched mRNA transcription and protein expression levels were observed in fractional members of MAP relevant genes, and mRNA expression of MAP members was associated with cancer stages in NSCLC patients. Higher transcription levels of MAP1/1S were significantly correlated with shorter OS in NSCLC, while higher mRNA expression levels of MAP2/4/6/7/7D3 exhibited superiority in OS of NSCLC patients. Besides, the 50 frequently altered neighbor genes, which were significantly correlated with MAP mutations, and associated network were further analyzed and constructed. This study revealed that the Hippo signaling pathway relevant genes, such as DLG1, DLG2, DLG3, DLG4, RASSF1, and GSK-3β, were significantly associated with MAP alterations. Moreover, the results from GO enrichment analysis and KEGG analysis revealed that the BPs such as GO: 0022604 (regulation of cell morphogenesis), CCs such as GO: 0005874 (microtubule), MFs such as GO: 0015631 (tubulin binding), and pathways such as has: 04390 (Hippo signaling pathway) were remarkably regulated by the MAP mutations in NSCLC. Further analysis of TCIA showed that the expression levels of MAP1A/1B/1S/4/6/7D1/7D3 were closely related to the response to immunotherapy. Finally, this study verified MAP7/7D2 expression was higher in NSCLC samples than in paratumor tissues of 20 NSCLC patients, but MAP2/4/6/7D3 expression was the opposite by RT-qPCR.

Among the MAPs, the MAP1 family is the first group of microtubule lattice-binding structural proteins discovered in the body that can interact with actin and microtubules (34). In the human genome, the MAP1 family typically consists of three members, namely, the MAP1A, MAP1B, and MAP1S (35). Recent studies have revealed that when exposed to cisplatin, increased autophagy level of LUAD cells was detected by Western blot analysis of the autophagosome-associated light chain 3 of MAP1A/1B (36). Moreover, the MAP1B has been reported to be a new target in paraneoplastic neuropathy and has a high predictive value for small cell lung cancer (5). In fact, knockdown of MAP1B-LC1 can also decrease cell migration and invasion during EMT in A549 cells (19). And similarly, MAP1S can bridge autophagic components with microtubules and mitochondria in both autophagosomal biogenesis and degradation (37). Another related article shows that the increased expression level of MAP1S could trigger autophagy, thereby inhibiting genomic instability to inhibit tumors (38). The present report indicated that the mRNA transcription level of MAP1A/1B/1S in NSCLC specimens was lower than the transcription level of normal specimens. Furthermore, through GEPIA, the results revealed that there was no significant correlation between mRNA transcription levels of MAP1A/1B/1S and the clinical stage of NSCLC patients. Furthermore, the MAP1 family relevant prognostic prediction of NSCLC patients was determined utilizing the Kaplan–Meier plotter. In NSCLC patients followed for 200 months, higher MAP1A and MAP1S expressions were significantly associated with poorer OS. But to our surprise, there was no significant correlation between the mRNA transcription level of MAP1B and the OS of NSCLC patients.

MAP2 is widely found in neurons and neurogenic tumor cells (39), and its ability to interact with microtubules plays a critical role in neuronal morphogenesis, such as neurite initiation (40). MAP2 is one of the neuronal MAPs that controls the cargo transport in the pre-axonal filtering zone of neurons (41). It has been shown that MAP2 is a valuable diagnostic tool to recognize and diagnose low-grade neuroepithelial neoplasms (17). In fact, MAP2 has been shown to be specifically expressed in neuroendocrine carcinoma and relevant tumor cell lines, such as small cell lung cancer and neuroblastoma (17, 18). In the present study, the results indicated that the mRNA transcription level of MAP2 in NSCLC specimens was lower than the transcription level of normal specimens, and this mRNA transcription level was not associated with the clinical stage of NSCLC patients. Besides, the lower MAP2 mRNA transcription level in NSCLC was significantly associated with a shorter OS.

The expression of MAP4 is ubiquitously observed in non-neural specimens and plays a critical role in microtubule assembly processes in human cells. The researchers found a higher proportion of MAP4 to stathmin mRNA in NSCLC tissues than the above proportion in normal specimens, demonstrating that this proportion might be a potential prognostic marker in NSCLC patients (42). Furthermore, *in vitro* studies have shown that MAP4 knockdown can effectively prevent cancer cell migration and tumor invasion during tumor development in LUAD (16). Clinical statistical analysis also showed that MAP4 protein could accelerate tumor invasion and cancer cell migration, which are closely related to the progression of LUAD and poor prognosis (16). Another study showed that MAP4 had been identified as a potential prognostic marker for predicting the clinical efficacy of platinum-based chemotherapy in patients with NSCLC through proteomics analysis (7). In the present study, the results indicated that the mRNA transcription level of MAP4 in NSCLC specimens was lower than the transcription level of normal specimens, and this mRNA transcription level was not associated with the clinical stage of NSCLC patients. In addition, in all of the patients with NSCLC, a lower MAP4 mRNA transcription level was significantly associated with a shorter OS.

MAP6 is found highly expressed in several cells, such as neuron and skeletal muscle cells (43, 44). The deletion or abnormal expression of MAP6 gene can lead to a variety of diseases, such as schizophrenia and skeletal muscle dysfunction (44, 45). But, until now, there are still few studies on the correlation between the mRNA transcription level of MAP6 and the corresponding prognosis of NSCLC patients. In the present study, the results indicated that the mRNA transcription level of MAP6 was lower in NSCLC specimens than in normal specimens, but this mRNA transcription level was significantly correlated with the clinical stage of NSCLC patients. The lower mRNA transcription level of MAP6 was significantly correlated with shorter OS in all of the NSCLC patients. The RT-qPCR results verified that the expression level of MAP6 in NSCLC samples was relatively low compared with that in paratumor tissues, although it was not statistically significant, possibly because the sample size was not large enough.

Up to now, four different MAP7 subtypes have been discovered in the human genome, namely, MAP7, MAP7D1, MAP7D2, and MAP7D3 genes (46). Indeed, some studies indicate that MAP7 family proteins can directly or indirectly promote the binding process of Kalinin-1 with microtubules and contribute to the microtubule transport of cellular cargoes (46). Besides, other studies have shown that in cytogenetically normal patients with acute myeloid leukemia (AML), patients with high mRNA transcription levels of MAP7 are correlated with adverse OS compared to those with low MAP7 expression (6). At the same time, MAP7 has also been reported to promote the EMT process of human cervical cancer cells by regulating the autophagy pathway and accelerate the tumor progression of cervical cancer (47). But the correlation between the mRNA transcription level of MAP7 family and the corresponding prognosis of NSCLC patients has not been reported. The results demonstrated that mRNA transcription levels of MAP7 and MAP7D2 were obviously increased in NSCLC tissues, while the mRNA transcription levels of MAP7D1 and MAP7D3 were reversed. Next, more enriched mRNA transcription levels of MAP7/7D3 were significantly associated with favorable OS of NSCLC patients. The RT-qPCR results verified that the expression level of MAP7D3 in NSCLC samples was relatively low compared with that in paratumor tissues, although it was not statistically significant, possibly because the sample size was not large enough.

Although this study explored the relationship between mRNA transcription levels and protein expression levels of different MAPs and associated prognosis in NSCLC patients, it should be noted that there are some limitations in this study. Firstly, there are a large number of MAP family members, but not all of them are included in each database, so this study only discusses MAP members that exist in each database. Secondly, the expression data of MAPs in this study are derived from diverse literature and databases, so the research results may be affected by selection or information bias. Therefore, we need further verification in more clinical and basic studies.

## Conclusions

In conclusion, this research systematically analyzed the correlation between the mRNA transcription level of MAP members and the corresponding prognosis/response to immunotherapy of NSCLC patients. The results of the present study revealed that more enriched mRNA transcription levels of MAP2/4/6/7/7D3 were observed to be prominently associated with favorable OS of NSCLC patients, while more enriched mRNA transcription levels of MAP1A/1S were associated with shorter OS. These results implied the high MAP1A/1S expression could serve as potential personalized therapeutic targets for patients with NSCLC, and the high MAP2/4/6/7/7D3 expression could serve as biomarkers for favorable prognosis in NSCLC. Moreover, the expression levels of MAP1A/1B/1S/4/6/7D1/7D3 were significantly correlated with IPS in NSCLC patients. Finally, the expression levels of MAP1A/1B/1S/4/6/7D1/7D3 were closely related to the response to immunotherapy.

## Data Availability Statement

The original contributions presented in the study are included in the article/**Supplementary Material**. Further inquiries can be directed to the corresponding author.

## Ethics Statement

The present research was ratified *via* the West China Hospital of Sichuan University Biomedical Research Ethics Committee and was carried out in accordance with the principles of the Declaration of Helsinki. Besides, all data in this study were derived from databases and published literature to ensure that all written informed consent had already been signed.

## Author Contributions

JL and QH are co-first authors, who contributed equally to this work. Study design: JL, QH, and HD. Data collection: JL, TT, ZT, YD, CC, and MG. Data reduction: JL, QH, HD, YQ, JZ, and XL. Results discussion and analysis: All authors. Article writing: JL. Manuscript modification: HD, MG, and XL. All authors contributed to the drafting and critical revision of the manuscript. All authors contributed to the article and approved the submitted version.

## Funding

This study was supported by the National Natural Science Foundation of China Program grant (81972607) and 1.3.5 project for disciplines of excellence, West China Hospital, Sichuan University (ZYGD20003).

## Conflict of Interest

The authors declare that the research was conducted in the absence of any commercial or financial relationships that could be construed as a potential conflict of interest.

## Publisher’s Note

All claims expressed in this article are solely those of the authors and do not necessarily represent those of their affiliated organizations, or those of the publisher, the editors and the reviewers. Any product that may be evaluated in this article, or claim that may be made by its manufacturer, is not guaranteed or endorsed by the publisher.

## Abbreviations

BP, biological process; CC, cell component; CIs, confidence intervals; DAVID, Database for Annotation, Visualization, and Integrated Discovery; EMT, epithelial–mesenchymal transition; GEPIA, Gene Expression Profiling Interactive Analysis; GO, Gene Ontology; HR, hazard ratio; KEGG, Kyoto Encyclopedia of Genes and Genomes; LC, lung carcinoma; LUAD, lung adenocarcinoma; LUSC, lung squamous cell carcinoma; MAPs, microtubule-associated proteins; MF, molecular function; NSCLC, non-small cell lung cancer; OS, overall survival; STRING, Search Tool for the Retrieval of Interacting Genes/Proteins; TCGA, The Cancer Genome Atlas; TCIA, The Cancer Immunome Atlas.
