# Supplementary material for: Expression of Microtubule-Associated Proteins in Relation to Prognosis and Efficacy of Immunotherapy in Non-Small Cell Lung Cancer
Source: Front Oncol. 2021 Oct 1;11:680402. doi: 10.3389/fonc.2021.680402 (PMC8517487; doi:10.3389/fonc.2021.680402)

Immunophenoscore: 5

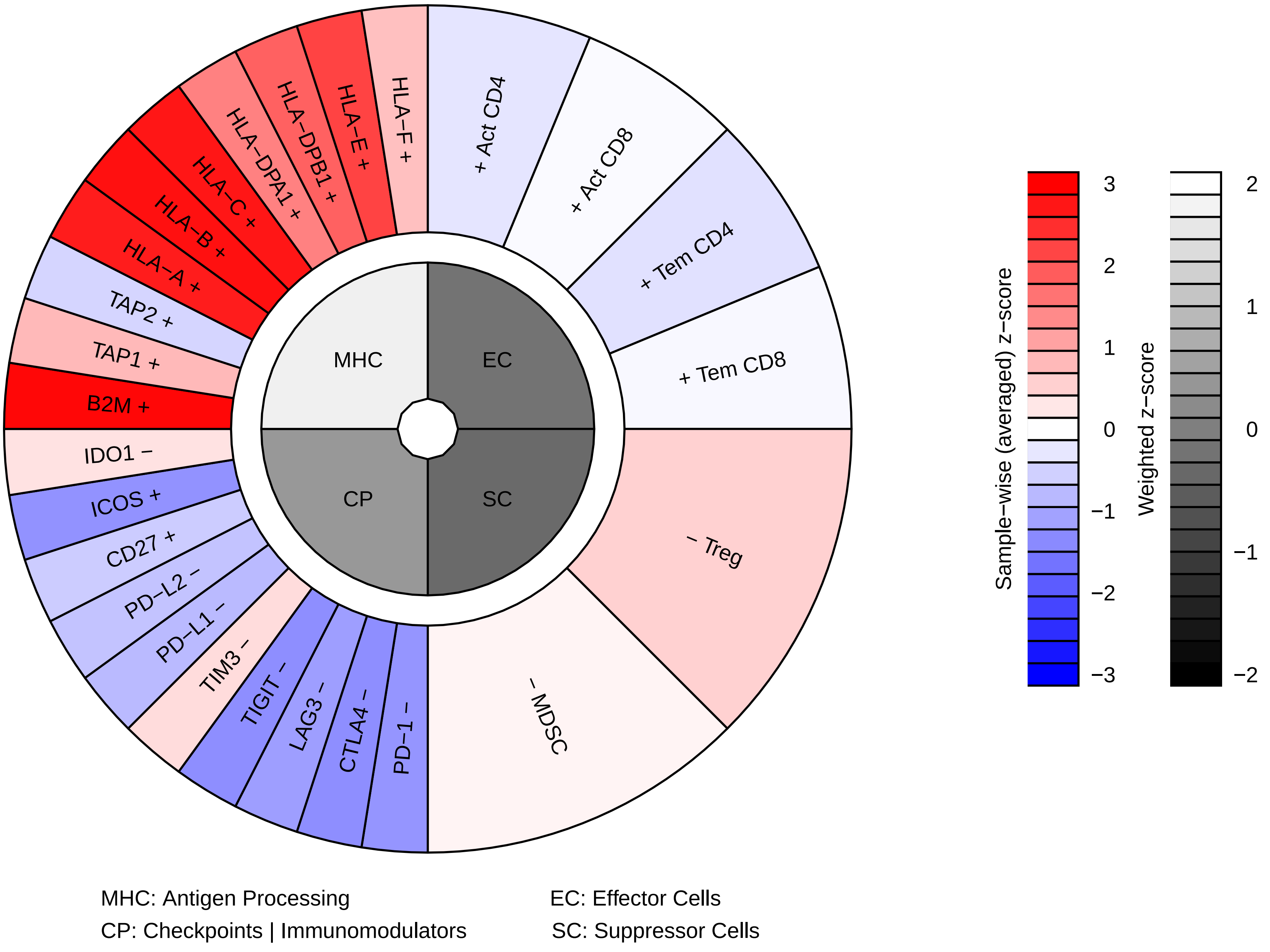

Supplement: Supplementary file 1 [file DataSheet_1.zip › immunephenotype/IPS_TCGA-05-5715-01A-01R-1628-07.pdf]
