# Supplementary figures and images for: Expression of Microtubule-Associated Proteins in Relation to Prognosis and Efficacy of Immunotherapy in Non-Small Cell Lung Cancer
Source: Front Oncol. 2021 Oct 1;11:680402. doi: 10.3389/fonc.2021.680402 (PMC8517487; doi:10.3389/fonc.2021.680402)

Immunophenoscore: 6

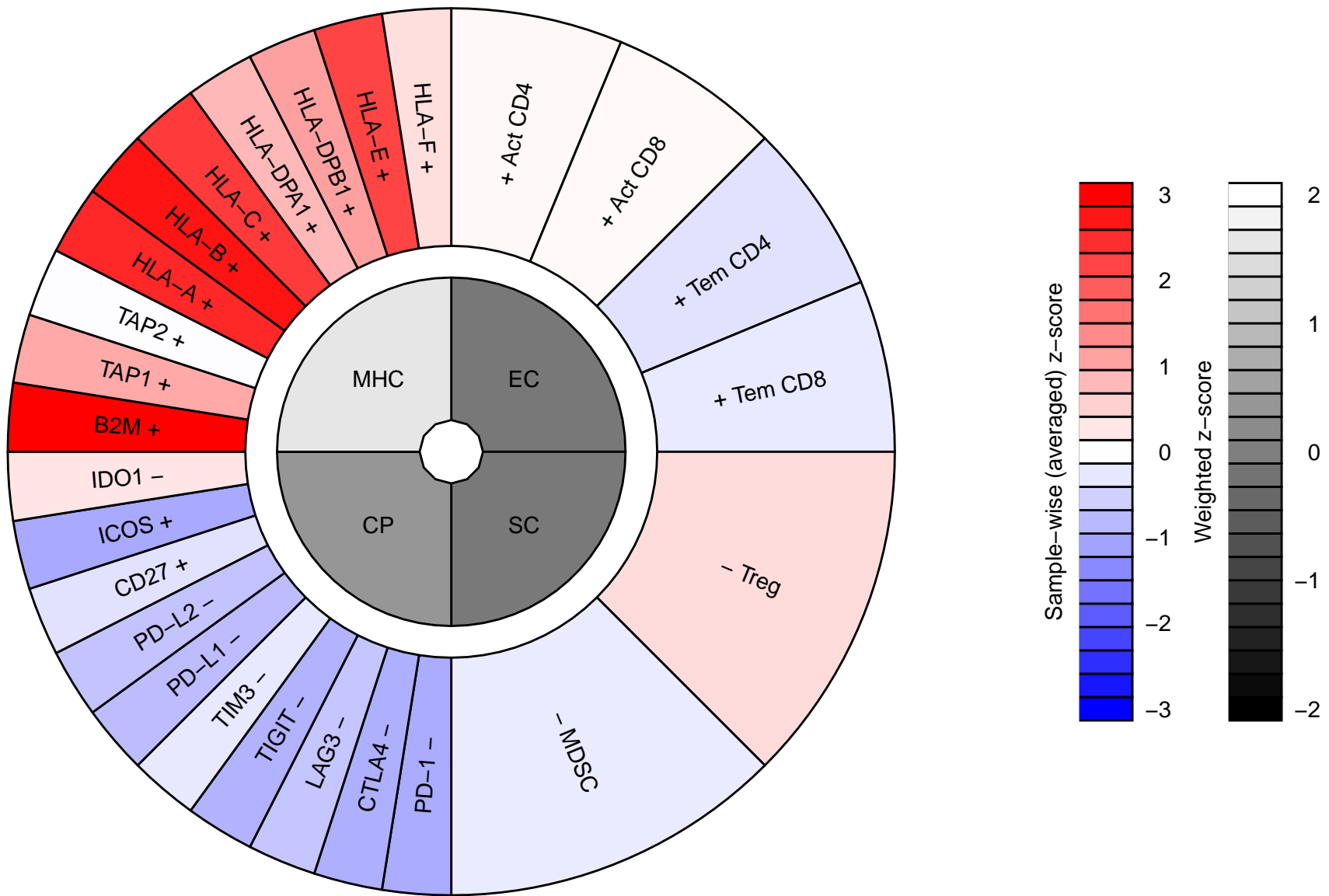

Supplement: Supplementary file 1 [file DataSheet_1.zip › immunephenotype/IPS_TCGA-18-3414-01A-01R-0980-07.pdf]

Immunophenoscore: 7

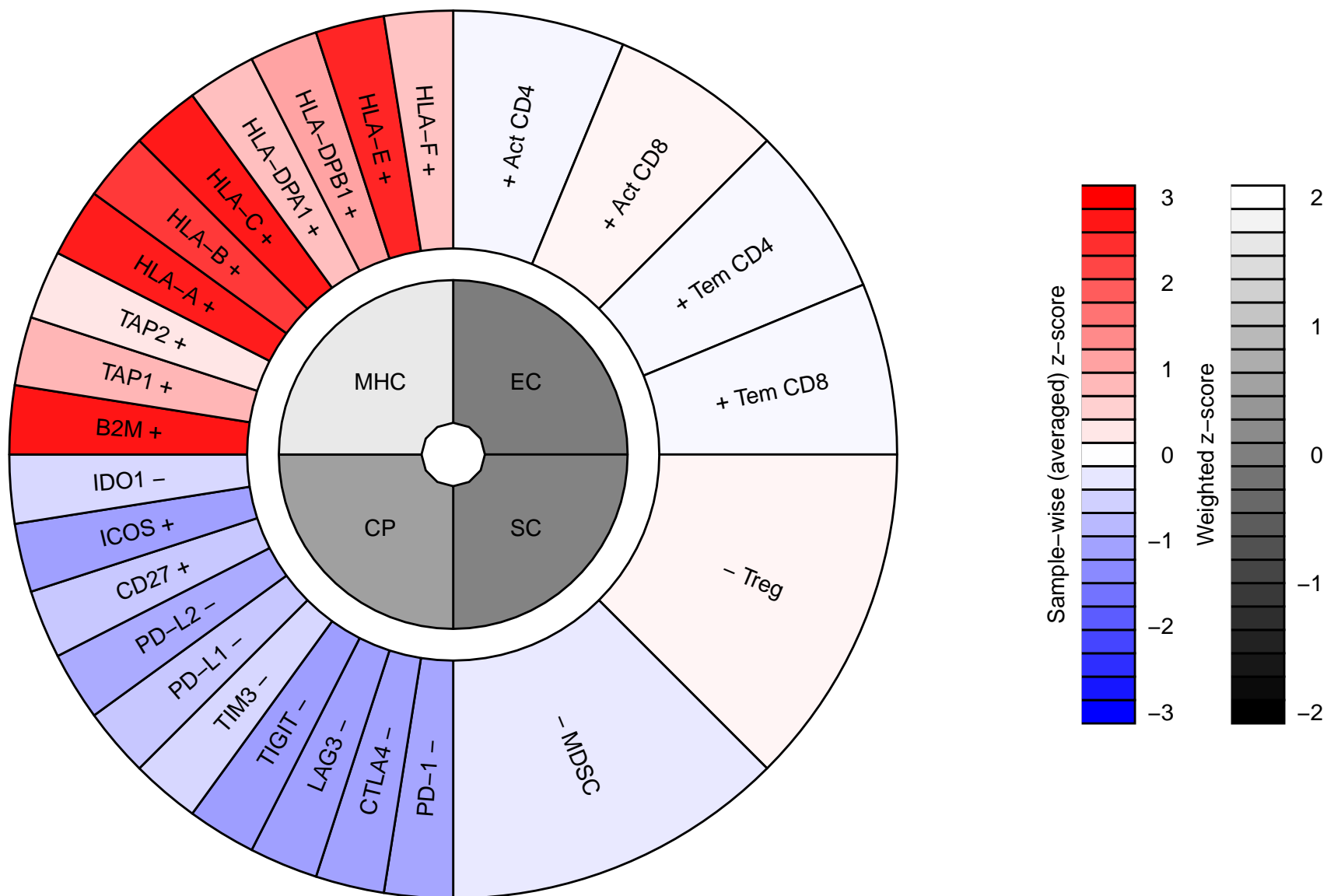

Supplement: Supplementary file 1 [file DataSheet_1.zip › immunephenotype/IPS_TCGA-21-1078-01A-01R-0692-07.pdf]

Immunophenoscore: 5

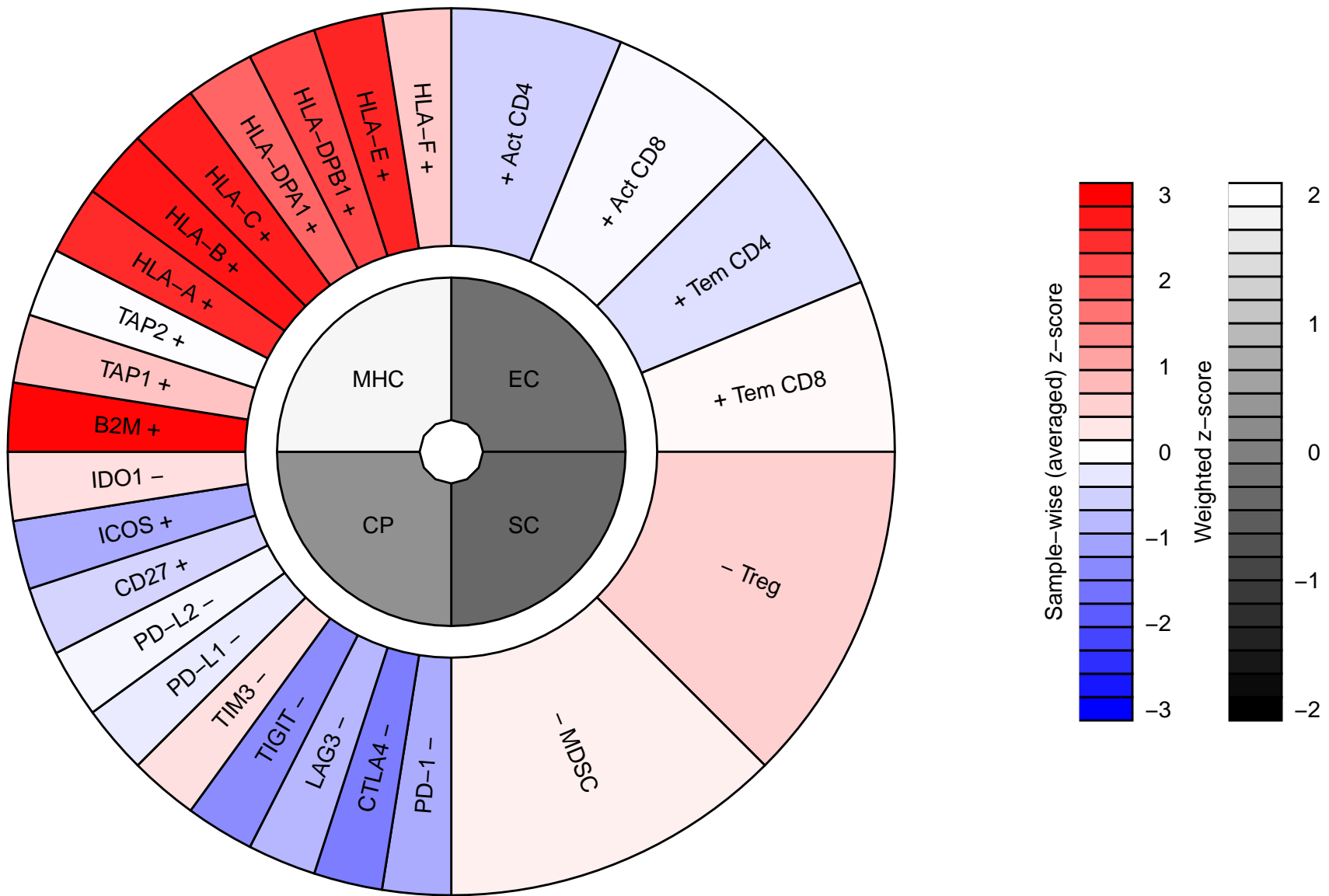

Supplement: Supplementary file 1 [file DataSheet_1.zip › immunephenotype/IPS_TCGA-22-5472-11A-11R-1635-07.pdf]

Immunophenoscore: 5

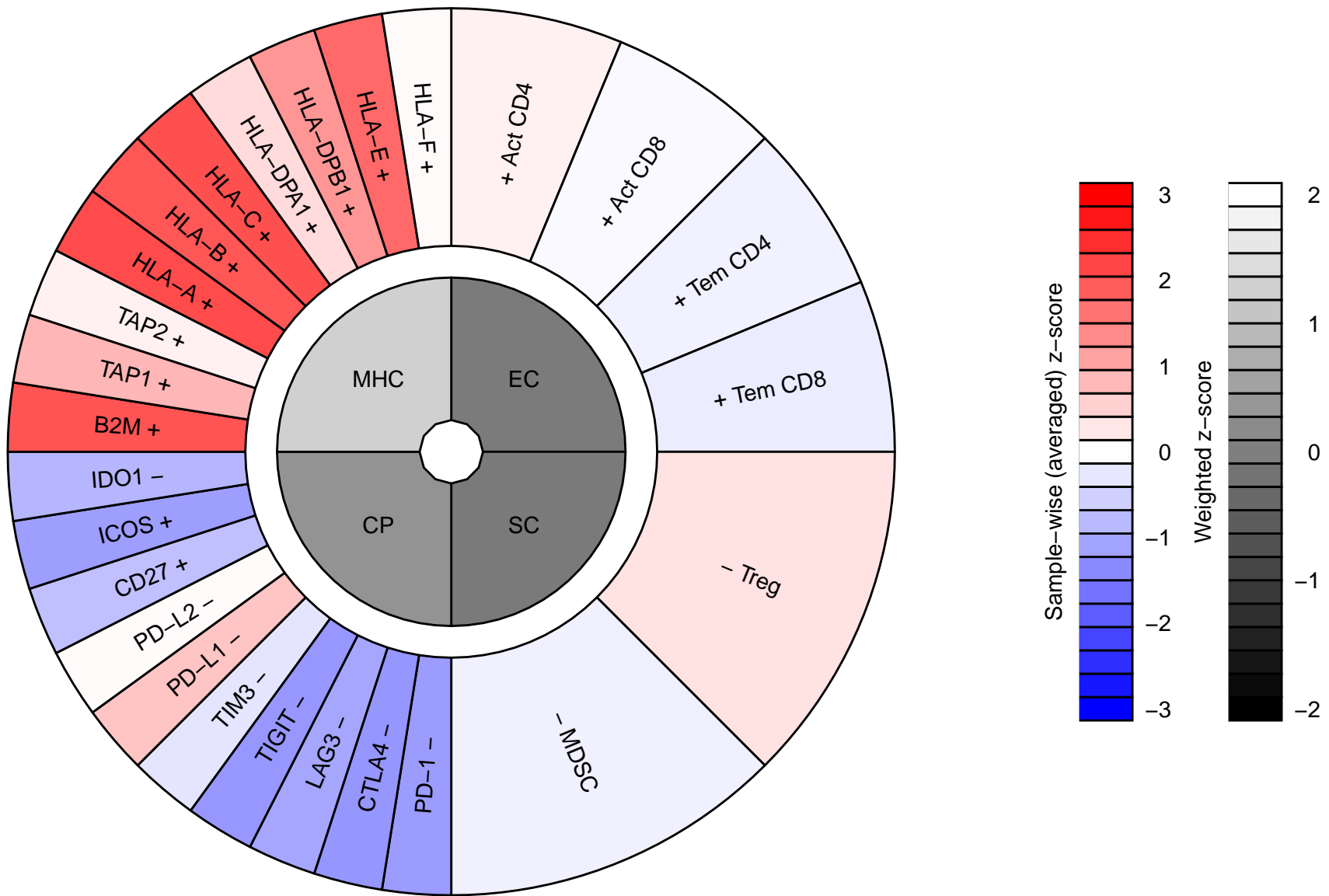

Supplement: Supplementary file 1 [file DataSheet_1.zip › immunephenotype/IPS_TCGA-33-4586-01A-01R-1443-07.pdf]

Immunophenoscore: 6

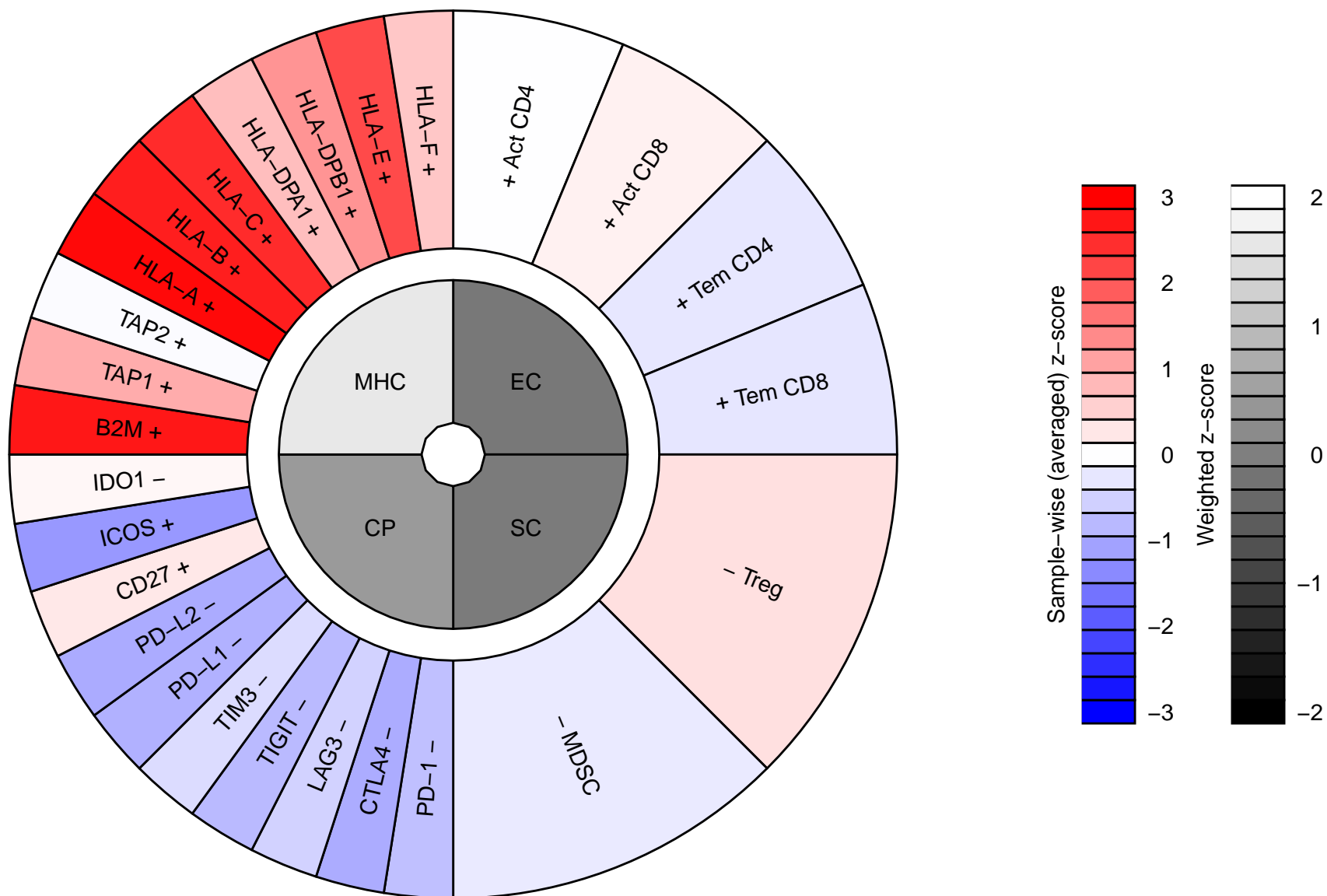

Supplement: Supplementary file 1 [file DataSheet_1.zip › immunephenotype/IPS_TCGA-33-6738-01A-11R-1949-07.pdf]

Immunophenoscore: 4

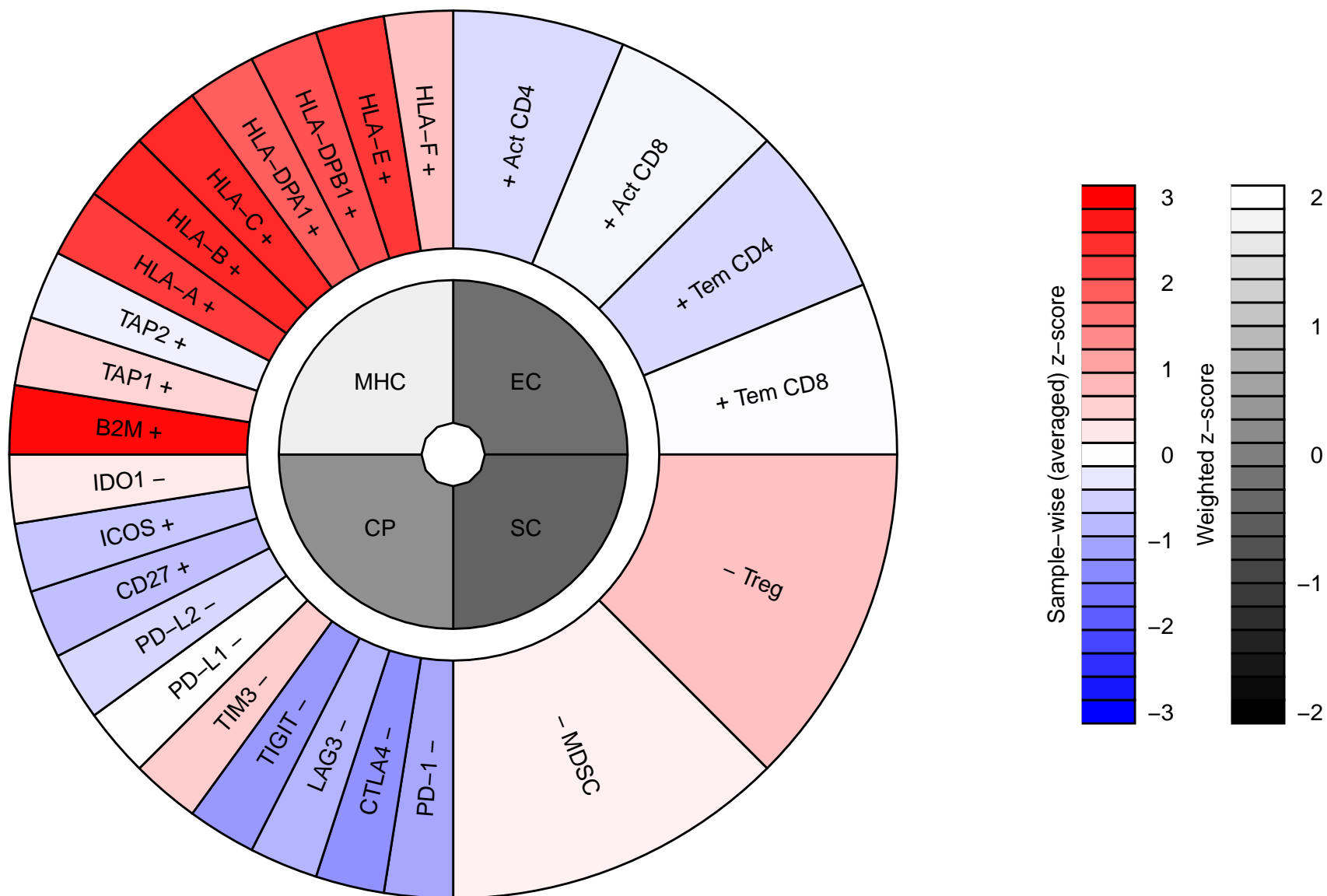

Supplement: Supplementary file 1 [file DataSheet_1.zip › immunephenotype/IPS_TCGA-38-4626-11A-01R-1758-07.pdf]
