# Supplementary material for: Expression of Microtubule-Associated Proteins in Relation to Prognosis and Efficacy of Immunotherapy in Non-Small Cell Lung Cancer
Source: Front Oncol. 2021 Oct 1;11:680402. doi: 10.3389/fonc.2021.680402 (PMC8517487; doi:10.3389/fonc.2021.680402)
Supplement: Supplementary file 3 [file DataSheet_3.zip › Supplementary Material 1.DOCX]

Supplementary Material

| **Target gene Primer sequence 5’-3’** | | |
| --- | --- | --- |
| MAP1A | Forward | TGTGCGTGTGCTTTTTCCAG |
|  | Reverse | CACAGGGTAACGCAGGAAGT |
| MAP1B | Forward | AAAGGTCAGCCCATCGAAGA |
|  | Reverse | TTGGGCGTCAGAGAGAAGTT |
| MAP1S | Forward | AGAAAGACCCCAAACCGAGT |
|  | Reverse | CCTGGGCATTCGTCTTCTTG |
| MAP2 | Forward | CTGGCACCCCACCAAGTTAT |
|  | Reverse | TGTGAGGGGTCCTGGGATAG |
| MAP4 | Forward | GTTGCAGTGGTGCAGAATGG |
|  | Reverse | AAGGCCTCTGCCTCTAGTGT |
| MAP6 | Forward | TTGCCATAGAGACGCAGCC |
|  | Reverse | CACCTTCCAGGCTCGGTAAT |
| MAP7 | Forward | AACATCCGCCCTGTCAAGAG |
|  | Reverse | CAGGTGTCCGCTCTTCAACT |
| MAP7D1 | Forward | CAACGTCTTAAAGCCGAGCA |
|  | Reverse | ACGTCTTCTTCACTGACCGT |
| MAP7D2 | Forward | CGAAAACTGGAAGAGCAGCG |
|  | Reverse | CCCGGAGCTTCTGTTTCCTT |
| MAP7D3 | Forward | AGCCCATCCTTGAGAGAGC |
|  | Reverse | CTCTGCGCTCTCTTGCTAATC |
| GAPDH | Forward | TCAAGGCTGAGAACGGGAAG |
|  | Reverse | TCGCCCCACTTGATTTTGGA |

**Supplementary Table1. The primer sequences for RT-qPCR.**


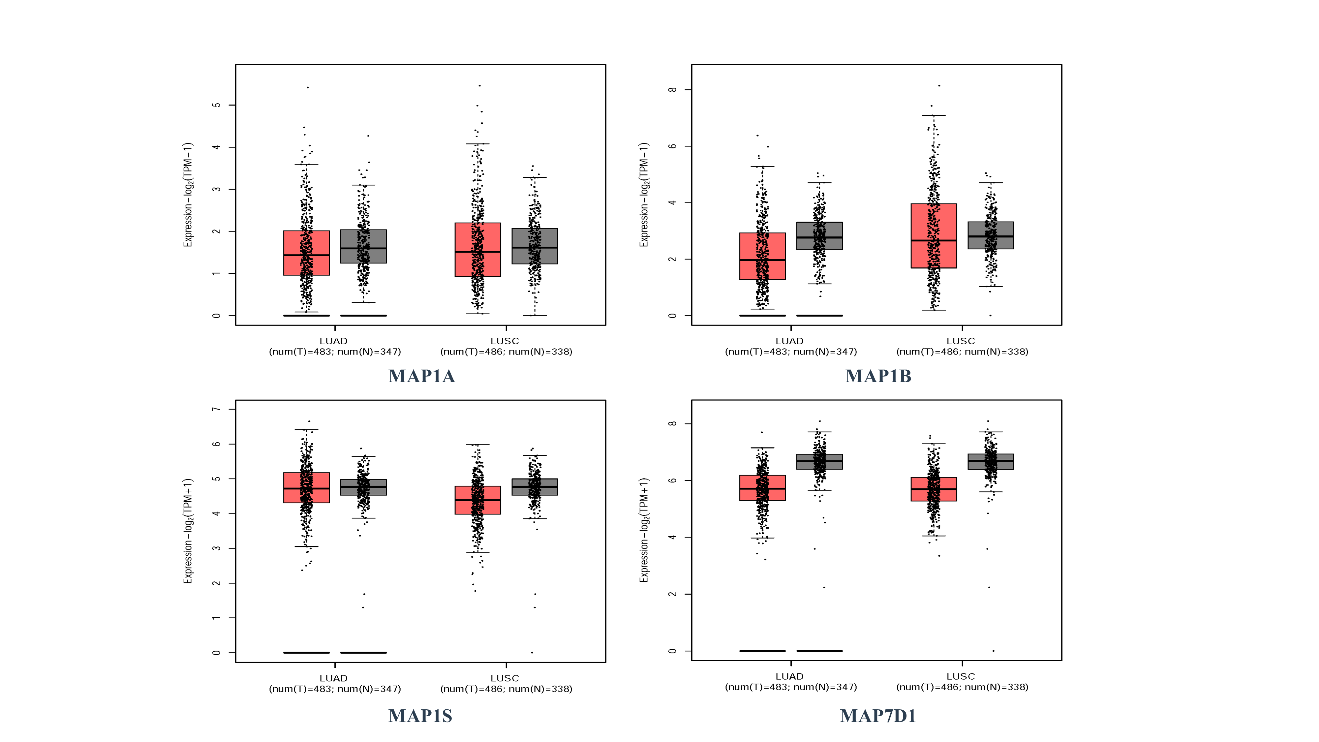


**Supplementary Figure 1. Transcription levels of distinct MAPs in LUAD, LUSC and normal lung specimens (GEPIA).** box plot. p>0.05.


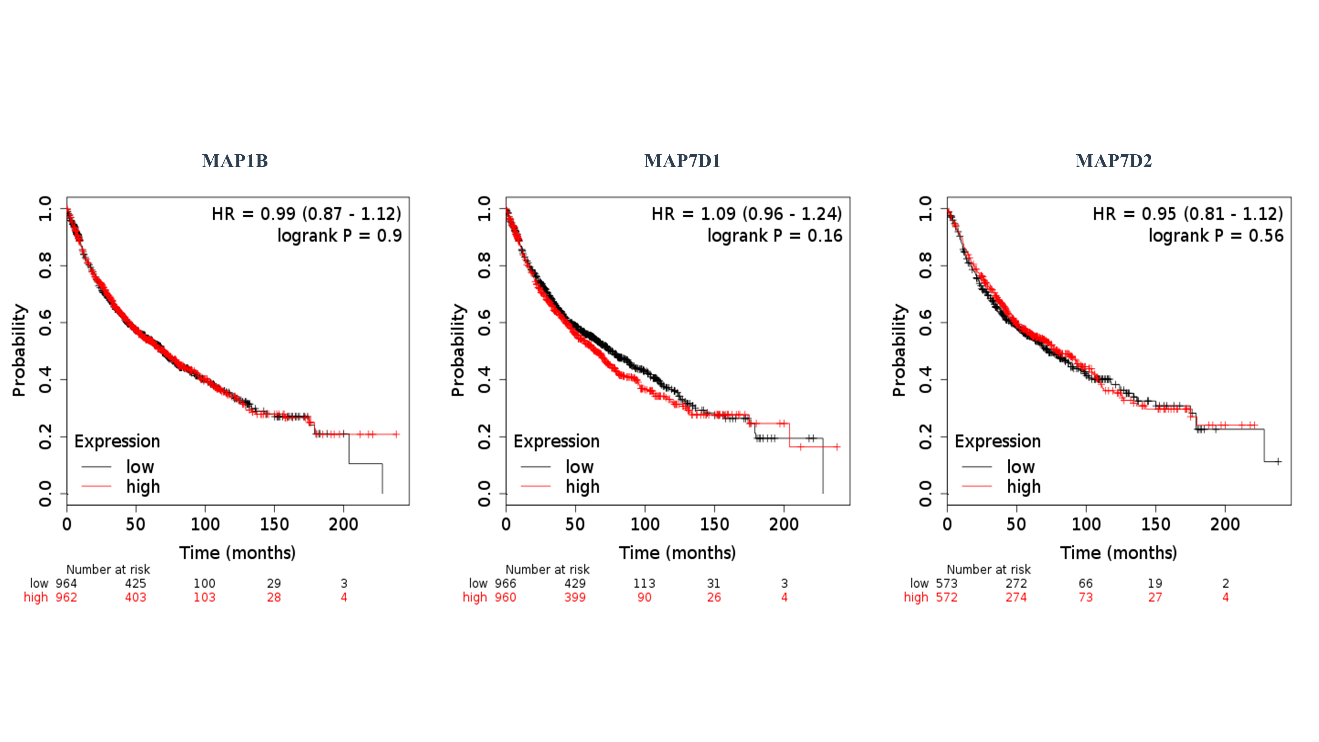


**Supplementary Figure 2. Kaplan–Meier survival curves stratified by 3 MAPs（p>0.05）, respectively.**


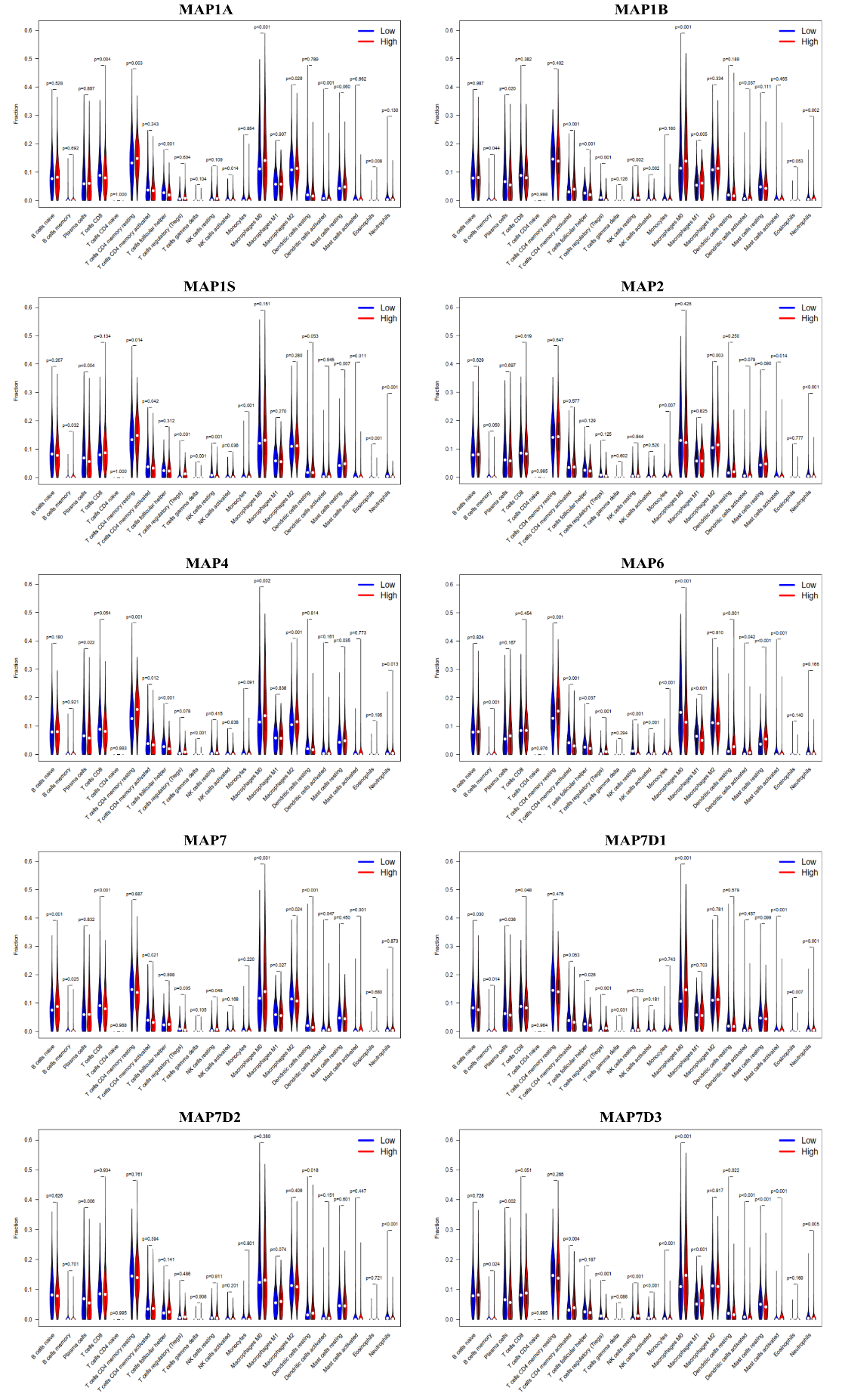


**Supplementary Figure 3. Immune cell infiltrations in high and low expression group according the median value of MAPs, respectively.**


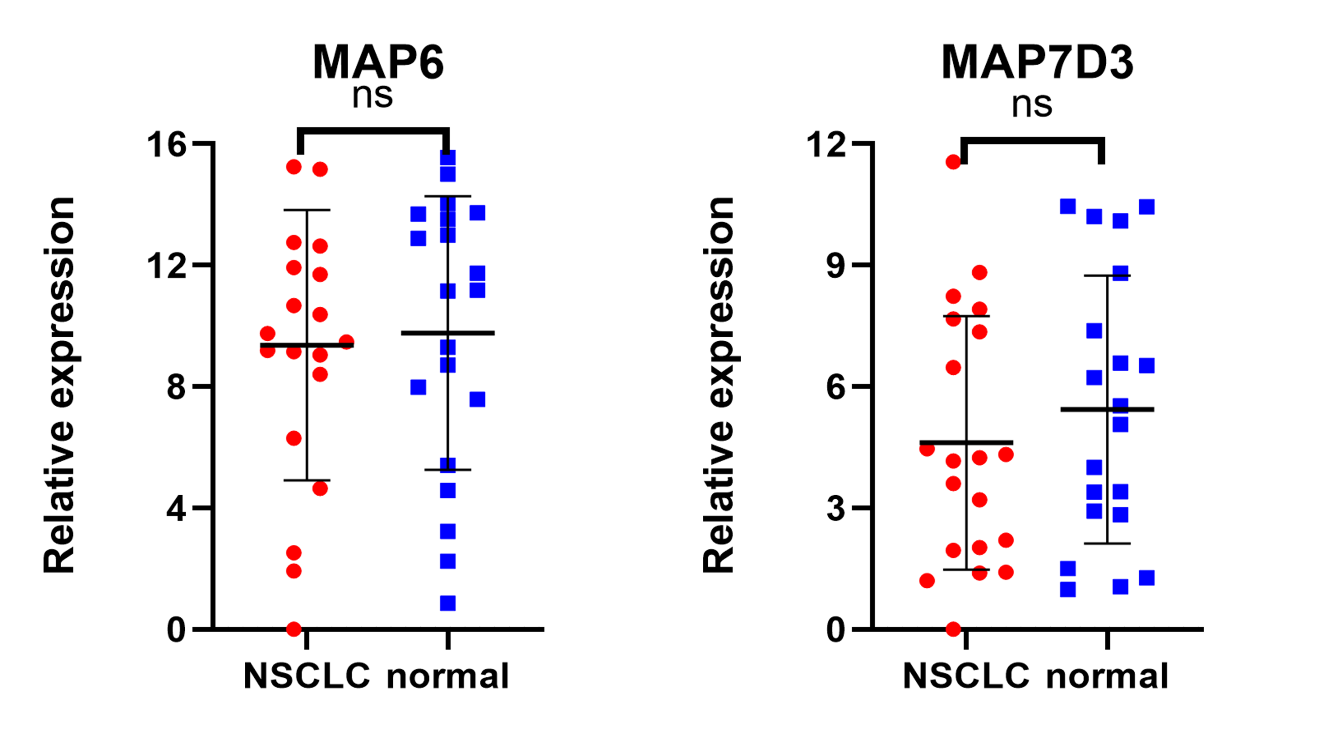


**Supplementary Figure 4. RT-qPCR analysis of MAP6/7D3 expression in 20 pairs of NSCLC samples and paratumor tissues. *P < 0.05 vs. normal lung tissues.**
